# Impulsivity-related predictors of adolescent substance use initiation

**DOI:** 10.1017/S0033291726103225

**Published:** 2026-02-06

**Authors:** Jodi Gilman, Kevin Potter, Jasmeen Kaur, Phil Lee, Randi Schuster, James Bjork, Alexander Weigard, A. Eden Evins, Joshua Roffman, Brenden Tervo-Clemmens

**Affiliations:** 1 Massachusetts General Hospital, USA; 2 Department of Psychiatry, Harvard Medical School, USA; 3 Virginia Commonwealth University, USA; 4 University of Michigan Medicine School, USA; 5 University of Minnesota, USA

**Keywords:** adolescence, alcohol, assessment, cannabis, impulsivity, nicotine, substance use initiation

## Abstract

**Background:**

Neurodevelopmental models regard impulsivity as a central risk factor for adolescent substance use. However, the practical utility of impulsivity in predicting substance use is complicated by variability among measures that encompass multiple methods and theoretical domains. Prior research has been constrained by cross-sectional designs, small sample sizes, and/or the use of a narrow subset of impulsivity measures.

**Method:**

Leveraging the ABCD dataset (*n* = 11,868), we identified and replicated correlations among impulsivity measures and assessed their prospective longitudinal and concurrent predictive utility regarding adolescent substance use outcomes before 15 years old. We then used simulation to inform how associations between impulsivity and substance use vary across sampling strategies (population vs. high-risk cohorts) and sample sizes.

**Findings:**

Correlations between questionnaire and behavioral measures of impulsivity were small, and questionnaires significantly outperformed behavioral measures in predicting substance use initiation, largely due to the contribution of the CBCL externalizing scale. Predictions of substance use based on impulsivity were statistically detectable but small according to clinical standards (AUCs 0.6–0.76), exhibiting sensitivity to sample size and base rate of substance use, and thus, poor absolute predictive performance. Large samples (*n* > 1,000) were needed to achieve adequate power for impulsivity measures to predict substance use initiation.

**Conclusion:**

These results support a significant but small contribution of impulsivity in predicting the onset of early adolescent substance use, indicating that these factors alone are insufficient for clinically deployable prediction. In community samples, large sample sizes are needed for reproducible impulsivity prediction of adolescent substance use.

## Introduction

Impulsive behavior often peaks during adolescence (Shulman et al., 2016; Tervo-Clemmens et al., 2024), a period marked by significant development in brain regions supporting self-regulation and decision-making (Casey & Jones, 2010). Although normal variation in impulsivity is related to core features of personality, such as openness and extraversion (Degnan et al., 2011), high levels of impulsivity, particularly among adolescents, are associated with socially inappropriate, maladaptive, and short-sighted behaviors (Sagvolden et al., 2005). Impulsivity is closely linked to substance use during adolescence, when increased experimentation with alcohol, nicotine, and cannabis is common (Chambers et al., 2003). Adolescents with higher impulsivity are more likely to initiate substance use early and engage in heavier use patterns than less impulsive peers (Castellanos-Ryan et al., 2011) and are less likely to perceive harms from substance use (Gilman et al., 2024). However, impulsivity is considered a multi-dimensional construct, with dozens of questionnaire and behavioral assessments. Therefore, understanding how different specific facets of impulsivity increase risk for adolescent substance use and eventual substance use disorder (SUD) is crucial for the development and refinement of interventions. For example, classroom-based educational interventions that are based on individual neurobehavioral traits (including impulsivity) have recently been shown to reduce adolescent drinking onset (Conrod et al., 2025).

Although higher impulsivity is cross-sectionally associated with greater substance use (Acton, 2003; de Wit, 2009; Krueger et al., 2002), the role of impulsivity as a prospective predictor of substance use is less clear (Tervo-Clemmens et al., 2020; Vergés et al., 2019). The multi-dimensionality and numerous available measures of impulsivity, which include parent and self-report scales (questionnaires), as well as cognitive and behavioral tests, complicate the reliable prediction of substance use. Various conceptual definitions of impulsivity exist, capturing different facets of the construct across measures, and they are often conflated in the literature (Cyders & Coskunpinar, 2011; Strickland & Johnson, 2021). Theories of impulsivity encompass a spectrum of processes that range from fully distinct to largely overlapping (Castellanos & Tannock, 2002; Reynolds et al., 2004; Reynolds & Schiffbauer, 2004; Swann et al., 2002), especially in relation to substance use (Vassileva et al., 2025). This assessment and theoretical heterogeneity poses challenges for clarifying the validity, reproducibility, and relative utility of adolescent impulsivity metrics as predictors of subsequent health outcomes. Such complexity is further compounded by statistical power and measurement limitations due to small sample sizes, narrow subsets of impulsivity measures, or both.

Impulsivity assessments include two major classes: questionnaires (e.g. Urgency-Premeditation-Perseverance-Sensation Seeking-Positive Urgency [UPPS-P] (Magid & Colder, 2007); ‘externalizing’ Child Behavioral Checklist (CBCL) (Achenbach, 1999), Behavioral Inhibition System/Behavioral Activation System (BIS/BAS) (Carver & White, 1994), and behavioral tasks (e.g. delay discounting task [DDT] (Ainslie, 1975; Logue, 1988; Rachlin & Green, 1972; Rachlin et al., 1991); the stop-signal task [SST] (Logan et al., 2014); NIH Toolbox flanker task [FT]). Assessment differences may allow for a nuanced understanding of a general construct of impulsivity (Reynolds et al., 2008; Sharma et al., 2014) but also pose interpretive and theoretical challenges. Assessment and theoretical differences make it difficult to compare results across studies, synthesize findings in meta-analyses, and develop clear, actionable information about how impulsivity relates to substance use and other health outcomes. Further complications arise from the often poor correspondence between various measures of impulsivity, particularly between questionnaires and behavioral tasks (Dang et al., 2020). Within this context, some have gone so far as to reject the notion of impulsivity as a psychological construct altogether (Strickland & Johnson, 2021). Still, others contend that impulsivity is indeed a stable, measurable, and predictive psychological trait (Huang et al., 2024). Therefore, although impulsivity measures are frequently integrated into substance use research, critical questions remain unresolved for clinical and developmental scientists. For instance, should research or prevention-focused settings use questionnaires (Woicik et al., 2009) or computerized behavioral tasks to measure impulsivity? (Caselles-Pina et al., 2023).

In this study, we adopt a working definition of impulsivity as a family of related personality and behavioral traits that reflect difficulties with self-regulation, reward processing, and inhibitory control. Rather than assuming a single unitary construct, we treat impulsivity as multidimensional, encompassing both trait-like self-reports and performance-based task measures. This framework allows us to directly compare how commonly used operationalizations, whether overlapping or distinct, predict adolescent substance use initiation and perceptions of harm.

Here, we investigate which assessments and domains of impulsivity most effectively predict the initiation of substance use during adolescence. To enhance insights on substance use risk, particularly among adolescents who have yet to initiate substance use, we also examined the relationship between metrics of impulsivity and the perceived harm of substance use across all participants. This is a known risk factor for substance initiation (Parker & Anthony, 2018a, b; Schleimer et al., 2019), including the initiation of nicotine/tobacco use in the ABCD sample (Doran et al., 2024). We use data from the Adolescent Brain Cognitive Development study (ABCD), a large (*n* = 11,868) multi-site, longitudinal study of adolescents to perform exploratory analyses following pre-registered guidelines (https://osf.io/fq4vz) and using pre-existing, matched discovery and validation subsamples (Feczko et al., 2021). We (1) identify correlations between questionnaire and behavioral measures of impulsivity in a longitudinal sample; (2) examine associations between baseline and concurrent impulsivity measures and substance use outcomes (substance use initiation and perception of harm from substance use); and (3) assess the sensitivity of these predictions across various sample sizes, from small, single-site studies to large multi-site consortia. Together, these analyses aim to refine multi-method and multi-domain theories of impulsivity and inform future translational research to address impulsivity-related adolescent health risks and substance use.

## Methods

The analyses used data from the ABCD study, a longitudinal research project involving 11,868 participants aged 9 to 11 years from 22 sites across the United States (Barch et al., 2018). Data were obtained from the NIMH Data Archive, Curated Annual Release 5.1. Details of inclusion and exclusion criteria are described elsewhere (Jernigan et al., 2018; Karcher et al., 2018). Most ABCD research sites ceded Institutional Review Board (IRB) approval to a central IRB at the University of California, San Diego, while the remainder obtained local IRB approval. All parents provided written informed consent, and all youths gave assent (Auchter et al., 2018).

We quantified impulsivity via three questionnaires and three behavioral tasks. Questionnaires included: the Behavioral Activation System (BAS) drive, fun-seeking, and reward responsiveness subscales; the Child Behavior Checklist externalizing subscale (CBCL-E); Urgency, Perseverance, Premeditation, and Sensation Seeking (UPPS) negative urgency, perseverance (lack of), premeditation (lack of), and sensation seeking, and positive urgency subscales. Because the CBCL-E includes items that explicitly ask about substance use, we removed those items when computing the subscale score to avoid inflated correlations and circular reasoning when predicting substance use outcomes. Behavioral tasks included: discounting rate estimated from the DDT—log transformed; the raw score from the NIH Toolbox flanker task (FT); and the stop signal reaction time estimated from the stop-signal task (SST) (Stop signal reaction time). See Supplementary Methods.

Adolescents were considered to have initiated substance use if they reported trying more than a sip of alcohol (not including religious sipping), more than a puff of nicotine, tobacco, or cannabis (including vapes), or use of any other substances (e.g. cocaine, etc.) at least once during years 0–3 (Sullivan et al., 2022).

The perceived harms of substance use total score was derived from questions assessing perceived risk of harm: ‘How much do you think people risk harming themselves (physically or in other ways) if they use [substance] regularly?’ (Question 1), ‘if they try [substance] once or twice?’ (Question 2), and ‘if they use [substance] occasionally?’ (Question 3). Response options were 0 = No Risk, 1 = Slight Risk, 2 = Moderate Risk, and 3 = Great Risk. Scores were summed across questions to yield a total ranging from 0 to 9, with higher scores indicating greater perceived risk. These questions were asked at follow-up years 1, 2, and 3. For nicotine/tobacco, the total ranged from 0 to 15 due to two additional items.

### Statistical analysis

We used established independent sets of participants via the ABCD Reproducible Matched Samples (ARMS), for split-half validation (Feczko et al., 2021). We used the ABCD ARMS-1 dataset (*N* = 5,770) as our discovery data and the ABCD ARMS-2 dataset (*N* = 5,757) as our validation data.

We focused on three sets of predictors of our outcomes of interest: Set A for base/demographic predictors, which included age and sibling status (single child, sibling, twin/triplet), biological sex, race, ethnicity, family income, parental education, parental employment, and parental substance use problems, parent mental health issues, and parent job/police issues; Set B for the questionnaire-based impulsivity measures (UPPS, BAS, and CBCL-E subscales); and Set C for behavioral-based impulsivity measures (delay discounting, SST, and the flanker task). Additionally, we examined two types of predictors for Sets B and C: first, predictors collected in years 0–1 (baseline), and second, predictors collected in years 2–3 (concurrent). We imputed missing values in predictors and our outcomes using predictive mean matching with all covariates and outcomes serving as predictors (see Supplementary Tables 1.1–1.2). Test statistics were pooled across imputations according to Rubin’s rules (Rubin, 1987).

We assessed correlations between the Set B questionnaire-based and Set C behavioral-based impulsivity measures using the non-parametric Kendall rank correlation coefficient (Kendall’s τ). *P*-values were adjusted for multiple comparisons through the Benjamini–Hochberg method (Benjamini & Hochberg, 1995). In the preregistration of this study (https://osf.io/fq4vz), we determined that correlations would be deemed significant at an effect size threshold of a bivariate *r*-equivalent of 0.08.

To develop predictive models for substance use initiation and the perceived harms score, we employed multilevel models, using a logistic regression model for substance use initiation and a binomial regression model for the perceived harms score to accommodate non-normal distributions and bounded ranges. All models included a site-varying intercept. To ensure stable parameter estimates (i.e. when using logistic regression with low base rates), we fit models using penalized maximum likelihood (see Supplementary Methods).

We examined associations between individual predictors and our outcomes of interest by fitting a full model with predictors for Set A, B, and C, allowing us to estimate effect sizes and statistical significance while accounting for between-predictor correlations. When evaluating the significance of effect sizes (odds ratios when predicting substance use initiation, change in percent total when predicting perceived harms), we adjusted for multiple comparisons using the Benjamini–Hochberg approach. We fit the full model separately to the discovery and validation data sets, and flagged predictors as statistically significant only if their effects replicated in the validation set (i.e. if the direction of the effect size was equivalent across data sets and adjusted *p* < .05 in all cases).

Additionally, we conducted model comparison tests to evaluate whether our sets of impulsivity measures improved out-of-sample predictive performance above and beyond our base predictors (Set A). This approach provides insight into the relative contribution of the different classes of impulsivity predictors on predictive performance. We first computed predictive performance for a model with only Set A, the base predictors. We then compared performance for this simpler, reduced model against three more complex models: (1) a model with Set A and B, assessing the overall contribution of questionnaire-based impulsivity measures, (2) a model with Set A and C, assessing overall contribution of the behavioral-based impulsivity measures, and finally (3), the full model with Set A, B, and C, assessing if there are even further improvements with the combination of both classes of impulsivity predictors. Out-of-sample predictive performance was assessed by fitting models to the discovery data set and evaluating their performance on the validation data set. We focus on predictive performance based on the area under the curve (AUC), using DeLong’s test to construct uncertainty intervals and to compare models (DeLong et al., 1988). For convenience and clarity, we also applied AUC for the binomial regression model, converting observed values and model predictions to binary values through a median split. We focus on AUC for predictive performance, because it is insensitive to issues such as low base rates (as is the case with substance use initiation). We also examined multiple alternative metrics of out-of-sample performance (positive predictive value and recall for substance use initiation, mean-square error for perceived harms, and we verified our model comparisons using 10-fold cross-validation rather than split-half validation (see Supplementary Methods).

To generate a power analysis, we used the substance use initiation outcome and generated 2,016 discovery and validation datasets through resampling. We used the fixed-effect estimates from the full model fit to the discovery dataset and, for each resampled dataset, generated a binary outcome using a standard logistic regression model. By adjusting the intercept of the generating model, we could examine power under two different rates of substance use (1) the rate observed in the ABCD sample (3.3%) and (2) a rate of 50% (e.g., for an enriched sample). We then refit univariate and multivariate models using the resampled predictors and simulated outcomes to estimate power. Our power analyses thus (a) are based on realistic data distributions (e.g. positively skewed impulsivity scores), (b) consider model misspecification (i.e. simpler models fit to data from more complex models), and (c) provide insight on the impact of low versus balanced rates of substance use.

### Preregistration

We submitted a preregistration to the Open Science Framework on 21 June 2022, prior to this data analysis, outlining our analytic and interpretative guidelines and validation strategies for this analysis. It included detailed descriptions of our research questions and hypotheses, instruments, analytical procedures, and our planned discovery and validation approaches and is available at https://osf.io/fq4vz. Please see the Supplementary Information for deviations from the preregistration.

## Results

**Figure 1.**
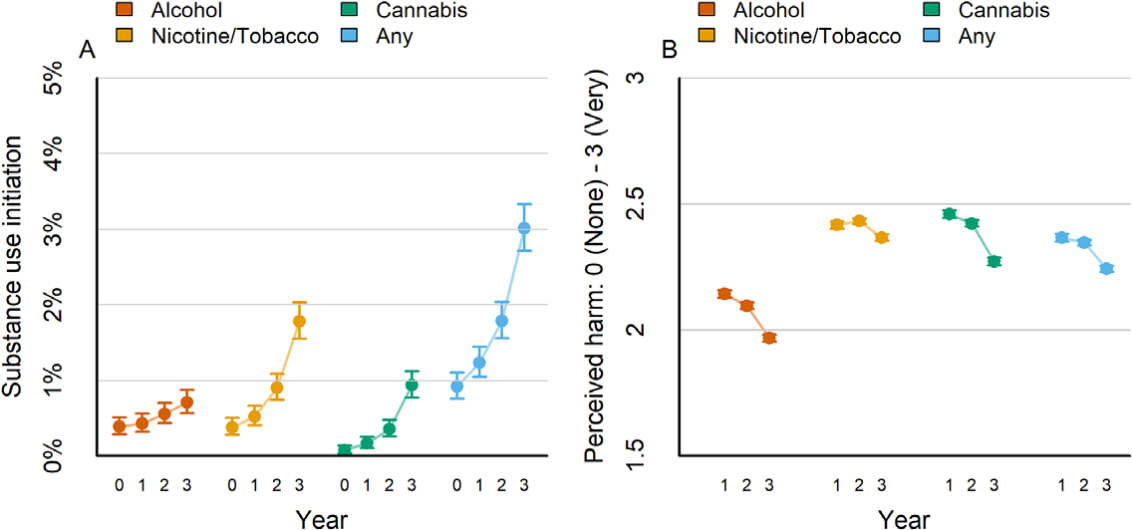
Developmental patterns of substance use initiation and perceived harm. (a) The proportion of adolescents who reported initiating substance use (see Methods) at each time point for alcohol (red), nicotine/tobacco (orange), cannabis (green), and any substance (blue) is presented. Initiation – defined as more than a sip of alcohol or more than a puff of nicotine/tobacco or cannabis – was carried forward from previous years. Proportions are calculated based on non-missing cases. Error bars represent 95% uncertainty intervals (see Methods). (b) The average perceived harm of alcohol, nicotine/tobacco, and cannabis, along with the overall average of the three, at each time point is displayed. The perception of harm was evaluated on a scale of 0–3, with 3 indicating the highest level of harm. Error bars indicate 95% uncertainty intervals using the T-distribution.

*From age 9 to 15 years, substance use initiation increases and perceived harm of substance use decreases.* This project analyzed data from 11,527 participants (Males: 6021, Females: 5506, age at baseline: 9.9 土 0.6) (see Supplementary Figure 1 for exclusions: *n* = 341, < 3%). Over the 4-year study period (participants ages 9–15), initiation of any substance, defined as first-time use of at least one full dose of alcohol, nicotine, cannabis, or other drug (see Supplementary Methods) showed a clear upward trend from approximately 0.9% (*n* = 109) at baseline to approximately 3.0% (*n* = 357) by study year 3 (Figure 1a). This substance use prevalence is lower than nationally representative estimates (9–12% of 8th graders; Miech et al., 2026) but similar to previous reports from the ABCD sample (Sullivan et al., 2022). Substance use initiation was highest for nicotine/tobacco, starting at 0.4% at baseline and increasing to approximately 1.8% by year 3 (Figure 1a). Perceived harm from substance use declined for all substances throughout the study period (Figure 1b), with cannabis demonstrating the largest decline in perceived harm.

**Figure 2.**
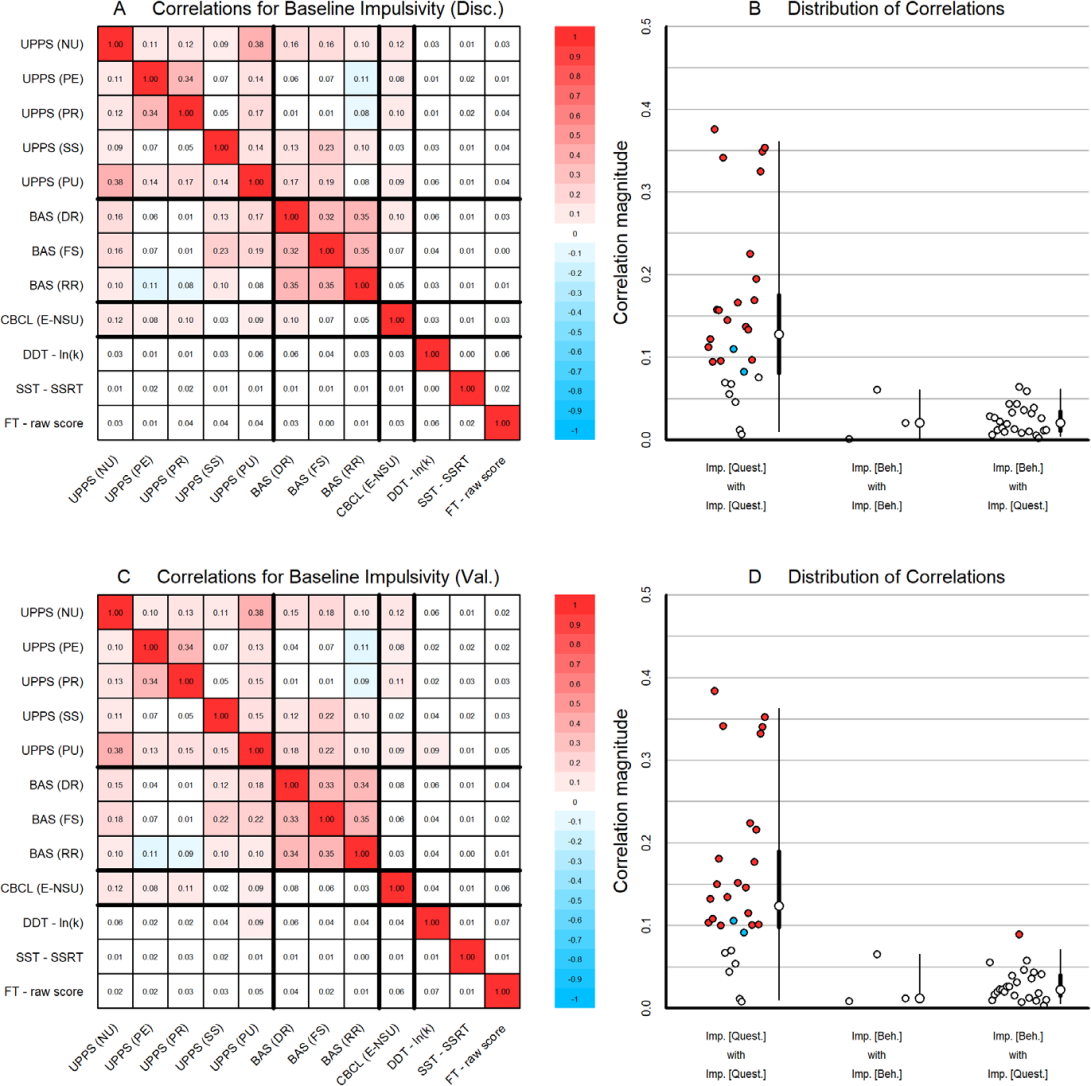
Correlations among impulsivity measures at the baseline assessment. (a) Correlation heatmap using Kendall’s *τ* for baseline impulsivity measures. Correlations with an absolute magnitude exceeding our pre-established threshold of .08 are shown in color (positive correlations: red, negative correlations: blue). Correlations below this threshold are shown in white. (b) Absolute magnitude of correlations (1) among questionnaire (Quest.) impulsivity (Imp.) measures, (2) among behavioral measures, and (3) between behavioral (Beh.) and questionnaire measures. Abbreviations: UPPS, Urgency-Premeditation-Perseverance-Sensation Seeking-Positive Urgency; NU, Negative Urgency subscale; PR Premeditation subscale; PE, Perseverance subscale; SS, Sensation Seeking subscale, PU, Positive Urgency subscale; BAS, Behavioral Activation System; DR, Drive subscale; FS, Fun Seeking subscale; RR, Reward Responsiveness subscale of the BAS; CBCL-E; the Externalizing (E) subscale of the Child Behavioral Checklist without substance use items; DDT – ln(k), the estimate of the log of the delay discounting rate; SST – SSRT, the estimate of the stop signal reaction time of the Stop Signal Task; FT, NIH toolbox flanker task; Imp, impulsivity; Quest, questionnaire; Beh, behavioral.

*Questionnaire and behavioral impulsivity are only weakly correlated.* First, we analyzed correlations between predictors from both Set B questionnaire-based impulsivity measures (UPPS, BAS, and CBCL-E) and Set C behavioral-based impulsivity measures (delay discounting, stop-signal task, and the flanker task). At baseline, subscales within each impulsivity questionnaire exhibited small to moderate statistically significant correlations among the other subscales of each questionnaire (UPPS; Kendall’s *τ* = 0.05–0.38, BAS *τ* = 0.3–0.35) (Figure 2), with nearly all surpassing our pre-established effect size threshold of a bivariate *r*-equivalent of 0.08 (see Methods). Modest correlations were also noted *between* subscales of different questionnaires (*τ* = 0.01–0.22), particularly the highest correlation between BAS fun-seeking and UPPS sensation-seeking. Behavioral-based impulsivity measures demonstrated weak correlations among themselves and with questionnaire-based impulsivity measures, which did not exceed our pre-established threshold. This pattern of results was replicated in the validation sample (Figure 2c,d). Similar correlation patterns were observed among the Year 3 measures (see Supplementary Figure 2). Additionally, replicating extensive prior research, we found a small but significant correlation between a lower perception of harm and an increased likelihood of substance use initiation (Kendall’s *τ* = 0.08, *p* < 0.001).

### Impulsivity questionnaires, but not behavioral tasks, weakly predict substance use initiation

**Figure 3.**
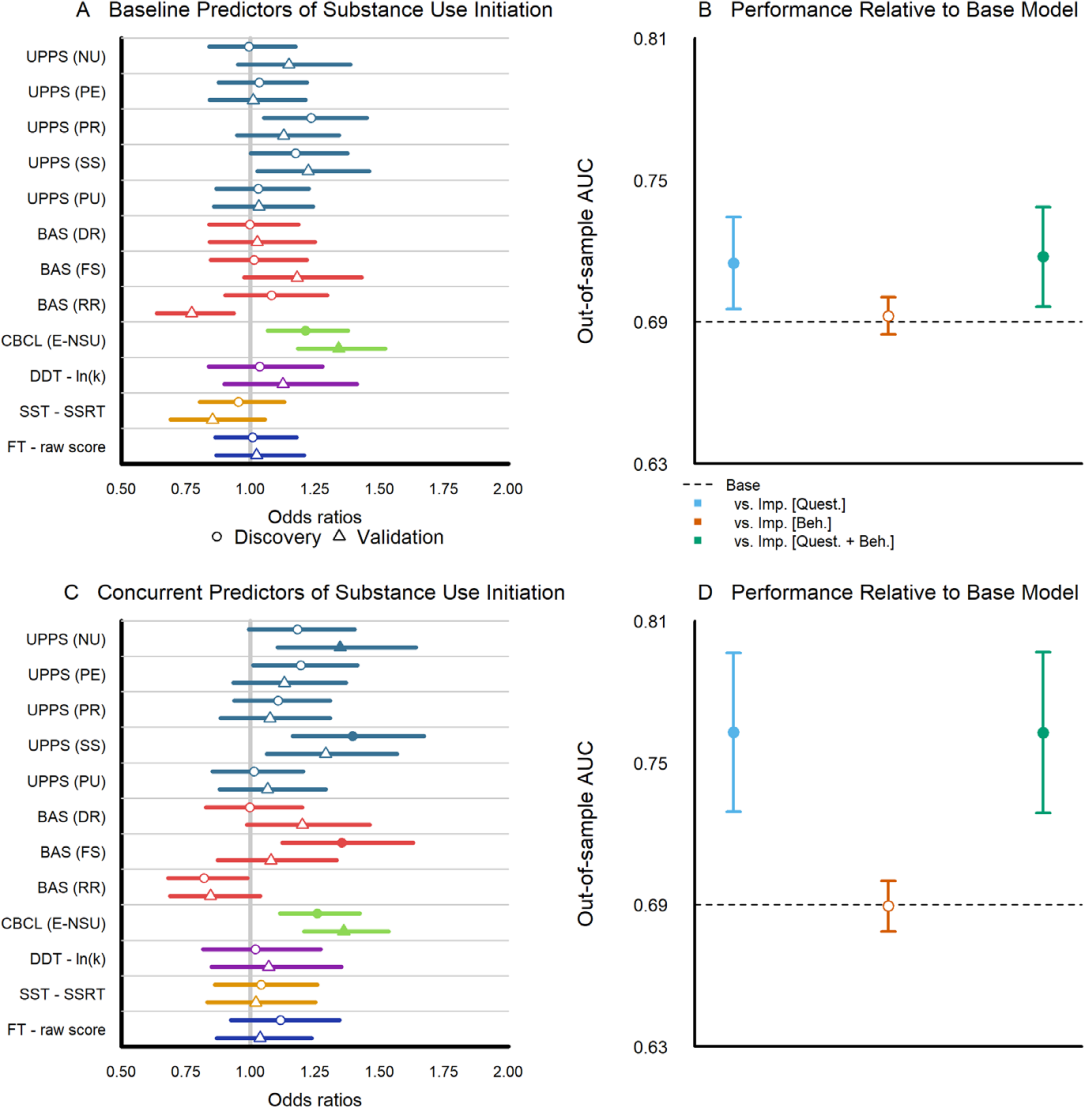
Impulsivity predictors of substance use initiation. (a) Odds ratios and 95% uncertainty intervals for each baseline impulsivity measure from the full model predicting any substance use initiation by year 3 (Circles represent estimates from the model fitted to the discovery data, while triangles denote estimates from the model fitted to the validation data; filled symbols indicate estimates with FDR-adjusted *p* < .05; see Supplementary Tables 2.1–2.4). (b) Area under the curve with 95% uncertainty intervals for baseline models fitted to the discovery data predicting substance use initiation based on the validation data; see Supplementary Table 2.5). (c) Odds ratios and 95% uncertainty intervals for each Year 2/3 (concurrent) impulsivity measure from the full model predicting any substance use initiation by year 3; see Supplementary Tables 3.1–3.4). (d) Area under the curve with 95% uncertainty intervals for concurrent models fitted to the discovery data, predicting substance use initiation from the validation data; see Supplementary Table 3.5.

*Effect sizes of individual baseline predictors.* We report odds ratios and 95% uncertainty intervals for discovery and validation data sets across Set B (questionnaire-based impulsivity measures) and Set C (behavioral-based impulsivity measures) predictors assessed at baseline (Years 0–1), as estimated from a comprehensive, multivariate model fit to substance use initiation by Year 3 using all three predictor sets (Figure 3a). Among all baseline impulsivity predictors, higher scores on the CBCL-E significantly predicted substance use initiation in both discovery and validation samples. The UPPS-P lack of premeditation scale served as a significant predictor in half of the data but did not replicate between discovery and validation samples. No behavior-based impulsivity measures predicted substance use initiation. Among Set A predictors, only older age significantly predicted substance use initiation by Year 3 in both discovery and validation samples (Supplementary Figure 3).

*Model comparisons examining sets of baseline predictors.* We report model comparisons of out-of-sample predictive performance (models fit to discovery data predicting substance use initiation from validation data) using area under the curve (AUC) for models using baseline (Year 0–1) predictors. We compare a reduced model with only Set A base predictors (e.g., general demographics like age, sex, etc.) against more complex models with predictors from (1) Set A and B (questionnaire-based impulsivity), (2) Set A and C (behavioral-based impulsivity), and (3) Set A, B, and C (full model) (Figure 3b). The reduced model with Set A predictors had an AUC of 0.688. The model with Set A and B predictors for questionnaire-based impulsivity measures had an improved AUC of 0.712 (*p* = 0.024), whereas the model with Set A and C predictors for behavioral-based impulsivity measures showed minimal improvement, with AUC = 0.690 (*p* = 0.534). The full model with Sets A, B, and C had the highest AUC of 0.715. We note that the predictive utility of all models remains notably low; the best-performing positive predictive value (PPV) for the full model was only 0.21% and recall was only 6.79% (Supplementary Table 2.5). This is in part driven by the extremely low base rate of substance use initiation of 3.3% and emphasizes the challenges in predicting complex and rare behavioral outcomes.

*Effect sizes of individual concurrent predictors.* We report odds ratios and 95% uncertainty intervals for discovery and validation data sets across Set B (questionnaire-based impulsivity measures) and Set C (behavioral-based impulsivity measures) predictors assessed concurrently (Years 2–3) (Figure 3c). Among concurrent impulsivity measures, higher scores on the CBCL-E once again predicted substance use initiation in both discovery and validation samples (Figure 3c). Results for Set A predictors are equivalent to findings for fit to the baseline predictors.

*Model comparisons examining sets of concurrent predictors.* We report model comparisons of out-of-sample predictive performance using AUC for models using concurrent (Year 2–3) predictors. We compare a reduced model with only Set A base predictors against more complex models with predictors from (1) Set A and B (questionnaire-based impulsivity), (2) Set A and C (behavioral-based impulsivity), and (3) Set A, B, and C (full model) (Figure 3d). The reduced model with Set A predictors had an AUC of 0.688. The model with Set A and B predictors for questionnaire-based impulsivity measures had an improved AUC of 0.760, *p* < 0.001, whereas the model with Set A and C predictors for behavioral-based impulsivity measures saw a slight worsening with AUC = 0.687, *p* = 0.978. The full model with Sets A, B, and C also had an AUC of 0.760. We note that the predictive utility of all models again remains notably low; the best-performing PPV for the full model was only 0.28% and recall was only 9.10% (see Supplementary Table 3.5).

### Impulsivity questionnaires, but not behavioral tasks, weakly predict perceived harms

**Figure 4.**
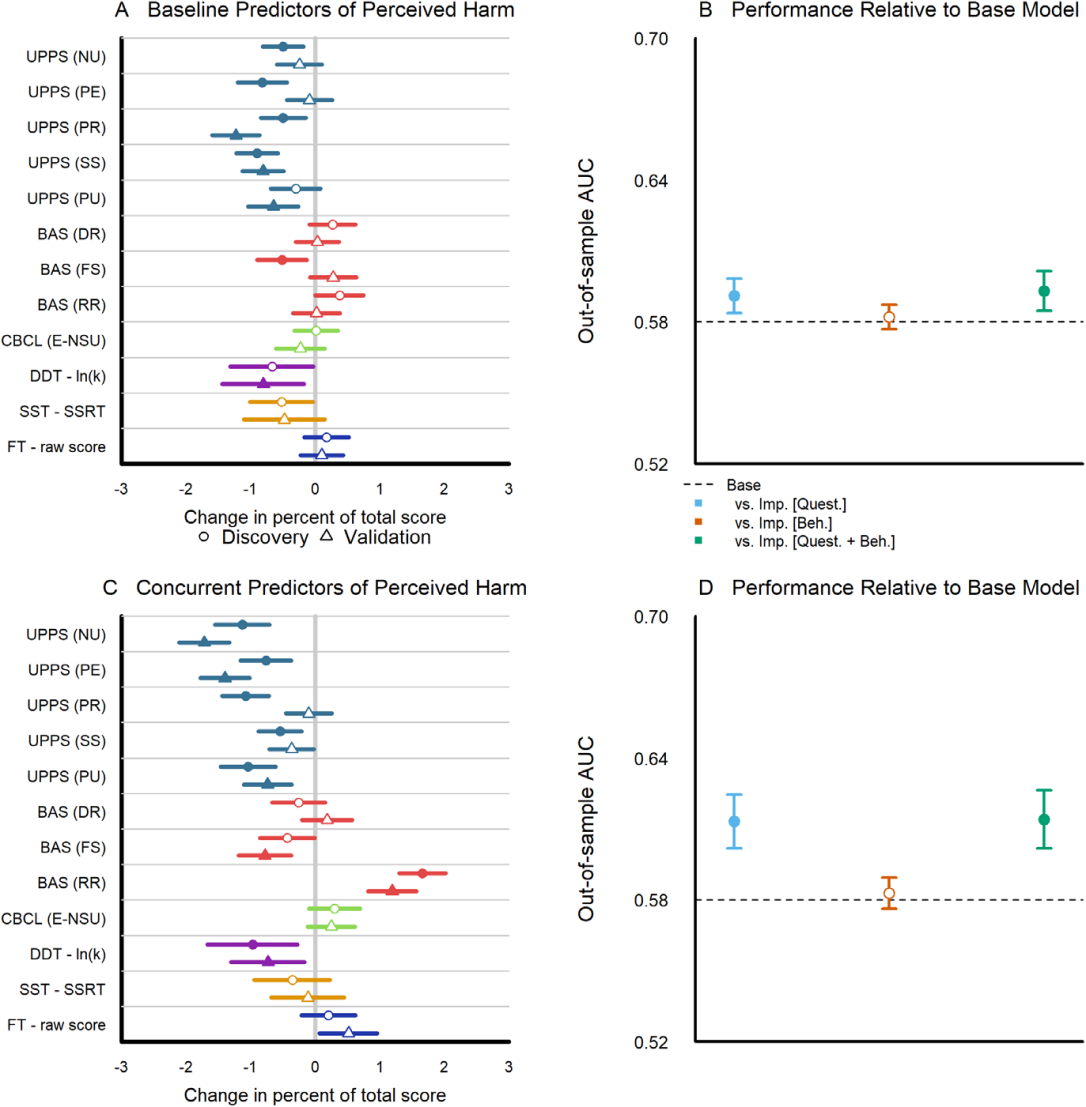
Impulsivity predictors of substance use perceived harm. (a) Change in percent total and 95% uncertainty intervals for each baseline impulsivity measure from the full model predicting total perceived harms at year 3 (Circles represent estimates from the model fitted to the discovery data, while triangles denote estimates from the model fitted to the validation data; filled symbols indicate estimates with adjusted *p* < .05; see Supplementary Tables 4.1–4.4). (b) Area under the curve with 95% uncertainty intervals for out-of-sample baseline predictors of perceived harm (models fitted to the discovery data predict a binary dichotomization of the total score using a median split from the validation data; see Supplementary Table 4.5). (c) Change in percent total and 95% uncertainty intervals for each Year 2/3 (concurrent) impulsivity measure from the full model predicting total perceived harms at year 3; see Supplementary Tables 5.1–5.4). (d) Area under the curve with 95% uncertainty intervals for out-of-sample concurrent predictors of perceived harm (models fitted to the discovery data, predicting a binary dichotomization of the total score using a median split from the validation data; see Supplementary Table 5.5).

*Effect sizes of individual baseline predictors.* We report change in percent total score and 95% uncertainty intervals for discovery and validation data sets across Set B (questionnaire-based impulsivity measures) and Set C (behavioral-based impulsivity measures) predictors assessed at baseline (Years 0–1), as estimated from a comprehensive, multivariate model fit to total perceived harm at Year 3 using all three sets (Figure 4a). Among all baseline impulsivity predictors, higher scores on the premeditation and sensation-seeking subscales significantly predicted lower perceived harms in both the discovery and validation samples. The negative urgency and perseverance subscales of the UPPS, the fun-seeking subscale of the BAS, and the log of the discounting rate in the delay discounting task were significant predictors in half of the data but did not replicate across discovery and validation samples. Among Set A predictors, only older age significantly predicted substance use initiation by Year 3 in both discovery and validation samples (Supplementary Figure 3). Among Set A predictors, older age, being female, and having parents reporting mental health issues all predicted lower perceived harm in both discovery and validation samples (Supplementary Figure 3).

*Model comparisons examining sets of baseline predictors.* We report model comparisons of out-of-sample predictive performance (models fit to discovery data predicting total perceived harms from validation data) using AUC for models using baseline (Year 0–1) predictors. We note that we compute AUC by applying a median split to the total score. We compare a reduced model with only Set A base predictors against more complex models with predictors from (1) Set A and B (questionnaire-based impulsivity), (2) Set A and C (behavioral-based impulsivity), and (3) Set A, B, and C (full model) (Figure 3b). The reduced model with Set A predictors had an AUC of 0.585. The model with Set A and B predictors for questionnaire-based impulsivity measures had an AUC of 0.596, the model with Set A and C predictors for behavioral-based impulsivity measures had an AUC of 0.587. While the model for Set A and B significantly differed from the reduced model, this was sensitive to choice of metric, since model comparisons using MSE only ranged from 40.6 to 40.9 and did not significantly differ (all *p* > .05; Supplementary Table 4.5).

*Effect sizes of individual concurrent predictors.* We report changes in percent total score and 95% uncertainty intervals for discovery and validation data sets across Set B (questionnaire-based impulsivity measures) and Set C (behavioral-based impulsivity measures) predictors assessed concurrently (Years 2–3) (Figure 4c). Among all concurrent impulsivity predictors, higher scores on the negative urgency, premeditation, and positive urgency subscales of the UPPS as well as the log discounting rate for the delay discounting task all significantly predicted lower perceived harms in both discovery and validation samples. Higher scores on the reward responsiveness subscale of the BAS predicted higher perceived harms in both discovery and validation samples. The sensation seeking subscale of the UPPS and the fun-seeking subscale of the BAS all served as significant predictors in half of the data but did not replicate between discovery and validation samples. Results for Set A predictors are equivalent to findings for fit to the baseline predictors.

*Model comparisons examining sets of concurrent predictors.* We report model comparisons of out-of-sample predictive performance using AUC for models using concurrent (Years 2–3) predictors. We compare a reduced model with only Set A base predictors against more complex models with predictors from (1) Set A and B (questionnaire-based impulsivity), (2) Set A and C (behavioral-based impulsivity), and (3) Set A, B, and C (full model) (Figure 3b). The reduced model with Set A predictors had an AUC of 0.585. The model with Set A and B predictors for questionnaire-based impulsivity measures had an improved AUC of 0.618, *p* < 0.001, while the model with Set A and C predictors for behavioral-based impulsivity measures showed minimal improvement with an AUC = 0.588, *p* = 0.461. Unlike baseline predictors, for concurrent predictors these findings were robust to choice of metric, with MSE exhibiting similar patterns of statistical significance (Supplementary Table 5.5).

*Contextualizing the prediction of impulsivity in adolescent substance use.* Across multiple outcomes in this large, multi-site study, we find evidence that impulsivity is a reproducible predictor of substance use, though generally with modest effect sizes and limited to a few measures. Even among questionnaire-based impulsivity, which outperformed behavioral assessments in all analyses (Figures 3 and 4), the odds ratios for substance use prediction are considered small by conventional standards (largest: CBCL-E 1.21, UPPS-P lack of perseverance 1.24). Such relatively small effects, coupled with complex population differences highlighted in large, multi-site studies like ABCD, may explain inconsistencies in prior research. To quantify this pattern and inform future research, our final analyses simulated the statistical power of questionnaire and behavioral impulsivity in predicting substance use across targeted smaller, single-site studies and multi-site population-based consortia.

**Figure 5.**
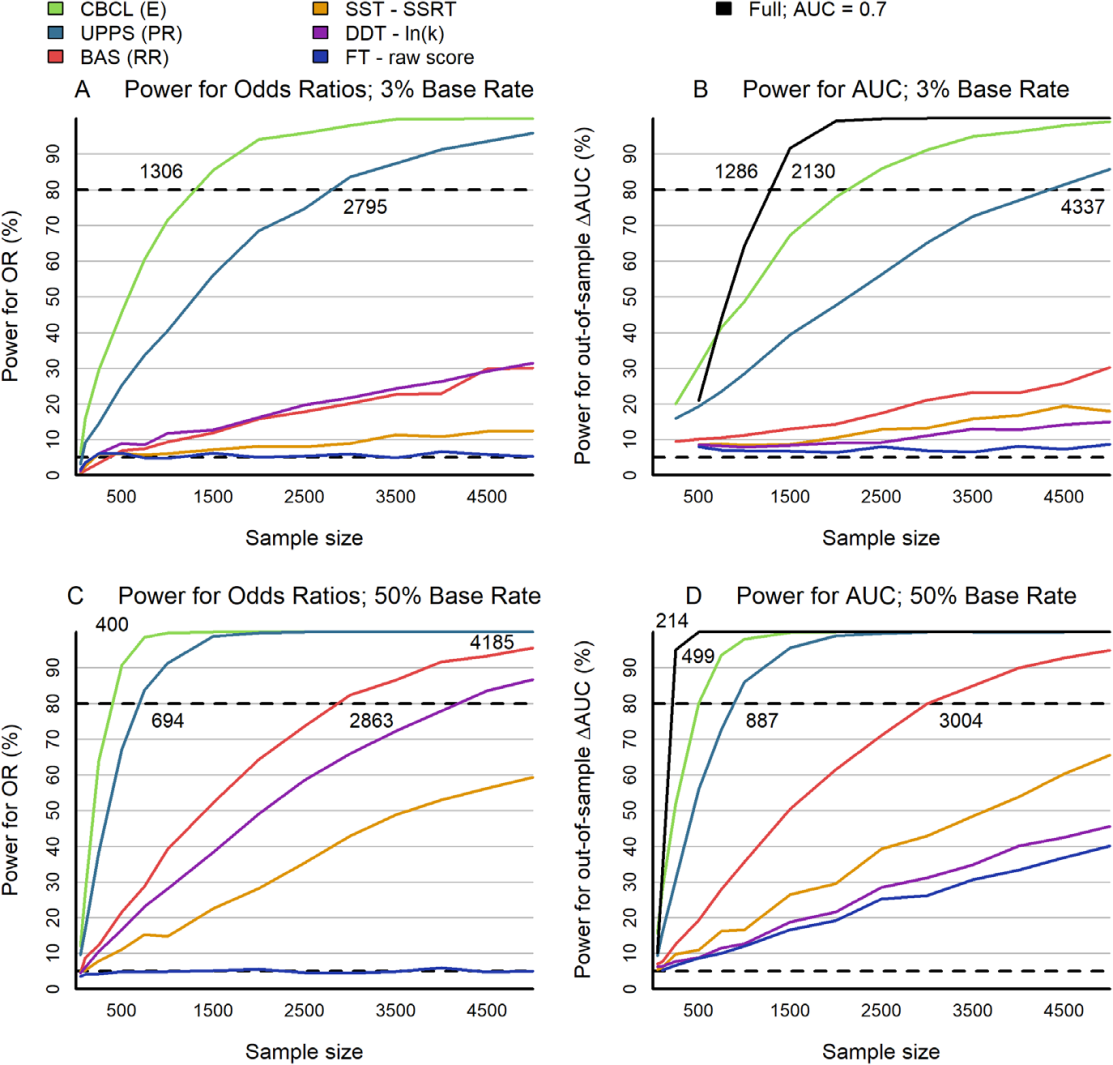
Power for within-sample odds ratios and out-of-sample AUC performance. Power estimates represent the percentage of significant (*p* < .05) results obtained from a Monte Carlo procedure with 2,016 repetitions. Simulation data were generated through resampling with replacement from the original ABCD data. All results are derived from logistic regression models that incorporate all predictors from the full model (see Methods), using coefficients set to point estimates from the full model fit to the original discovery data (cf, Figure 3). Power under varying base rates was determined by adjusting the value of the intercept in the generating model (see Methods). (a) Power to detect a significant odds ratio for the subset of best-performing predictors within each impulsivity measure, using a multivariate logistic regression fitted to all predictors for simulated discovery data assuming a base substance use rate of approximately 3%. (b) Power to detect a significant enhancement in AUC relative to an intercept-only model when predicting simulated outcomes from the validation data based on fits to the discovery data, given a substance use base rate of approximately 3%. Power is displayed for (a) the full model with all predictors (black) and (b) for reduced models that only include the best-performing predictor within an impulsivity measure. (c) Same as (a) but with a base rate of 50%. (d) Same as (b), but with a base rate of approximately 50%.

The largest effect sizes (odds ratios) for impulsivity-based predictors of substance use ranged from 1.01 to 1.25 (considering the subscale with the highest OR). Like any predictive analysis of a discrete outcome (such as substance use initiation by age 15), the ability to reproduce these effect sizes depends on the base rate. Given the 3% base rate of substance use initiation in the ABCD sample (by year 3), our data suggest that large samples are required for even the largest impulsivity effect sizes in similar population-based samples where substance use initiation is rare. The CBCL-E would necessitate the fewest number of participants (*n* = 1319) to meet standard power criteria of 80%, with p < .05, followed by the UPPS Premeditation subscale- (*n* = 2781) (Figure 5a). In contrast, effect sizes for other questionnaires and all behavioral measures queried in this study would be too small to be practical, even for large multi-site studies. Even with 5000 participants, BIS/BAS, SST, delay discounting, and Flanker have a power of only about 30% to detect substance use (Figure 5a). Out-of-sample prediction of substance use initiation (compared to the specific estimation of a given odds ratio) would also require large samples for statistical power with the ABCD sample’s base rate of 3% (Figure 5b). As expected from our prior analyses (Figures 3 and 4), a combined model incorporating all impulsivity measures demonstrated the highest power (Figure 5b), although it would require large samples (>1,000) for adequate power. Together, these power simulations illustrate the necessity of large multi-site studies to ensure reproducible prediction of substance use from impulsivity measures in extensive population samples of young adolescents where substance use initiation is infrequent.

Simulations also reveal potential power limitations of this large, multi-site study. Features of ABCD and other lab-based multi-site studies, including the requirement for in-person study visits and geographical constraints (in this case, sites that also have MRI facilities), may have contributed to what appears to be an underrepresentation of substance use (a base rate of 3% compared to national estimates of 9–12%). As predictive power is sensitive to the base rate of the condition of interest, analyses in ABCD and other studies with relatively low base rates will have less power than studies with a higher prevalence of substance use (all other factors being equal). For instance, simulations indicate that a balanced proportion of participants with and without substance use (i.e. a 50% base rate) would be significantly more powered in relatively smaller, investigator-initiated studies. Questionnaire impulsivity, but not behavioral, demonstrates adequate power in samples of several hundred participants (Figure 5c,d).

## Discussion

Determining how and which impulsivity measures best predict adolescent substance use is essential for developing accurate models of etiology, as well as prevention and intervention strategies. In this study, we analyzed data from the large, multi-site ABCD study to identify relationships among multi-method and multi-domain impulsivity measures and to assess the extent to which these measures, individually or collectively, predicted substance use initiation or perceived harm. We find that (1) correlations between questionnaire and behavioral measures of impulsivity were uniformly small and did not exceed our preregistered effect size threshold of a bivariate *r*-equivalent of 0.08; (2) questionnaires, particularly driven by the CBCL externalizing scale, outperformed behavioral measures in predicting both substance use initiation and harm perception, with nearly zero incremental benefit from behavioral measures compared to self-report; (3) due to the relatively modest prediction of substance use from all impulsivity assessments, future studies must account for sample sizes and substance use prevalence rates to ensure translational utility; and (4) overall, the *absolute* predictive capability for impulsivity predicting early substance use initiation is low, indicating that impulsivity, whether assessed via self-report or laboratory tasks, may not provide prediction accuracy at the threshold needed for clinically deployable screening, prevention targeting, or individual-level risk stratification in young adolescents over and above demographic and contextual risk factors. Our findings should be interpreted within the context of our working framework, which treats impulsivity as a multidimensional family of traits and behaviors rather than a unitary construct. This approach allowed us to compare how commonly used questionnaire- and task-based operationalizations perform when predicting substance use outcomes, while acknowledging that these measures may capture overlapping but not identical processes.

Weak correlations between questionnaire and behavioral measures of impulsivity in adolescents align with prior research in adults, where, for example, these two classes of assessments tend to correlate at approximately 0.1, with slightly higher correlations involving delay discounting. (Cyders & Coskunpinar, 2011) This is despite the fact that extreme clinical groups, such as adults with SUD, typically show increased impulsivity in both questionnaire and laboratory performance compared to healthy control groups (Lee et al., 2019). Future analyses with subsequent waves of the ABCD study may yield stronger results as the behavioral repertoire of the cohort expands into late adolescence and emerging adulthood.

Multiple questionnaire measures were significant, although weak, predictors of substance use initiation and perceived harm. We note that although the observed effect sizes for impulsivity measures to predict substance use were generally small, this is not necessarily unexpected given the inclusion of well-specified covariates, such as parental substance use problems, psychopathology, and socioeconomic factors, each of which is strongly associated with adolescent substance use and also correlated with impulsivity.(Brennan et al., 2024) By adjusting for these powerful covariates, our models set a higher threshold for prediction and help avoid confounding. In this context, even modest effects of impulsivity are meaningful, as they represent variance explained above and beyond established risk factors. Among these relatively modest predictors, the parent-report CBCL externalizing subscale emerged as the strongest predictor of both future and concurrent substance use, highlighting the utility of this widely used questionnaire for future substance use research. Relying on parent-report or teacher-report assessments bypasses the lack of insight into their own behaviors (metacognition) that is often seen in youth with ADHD and other disorders characterized by impulsivity (Unver et al., 2022). Other questionnaire measures showed mixed results across analyses but collectively provided incremental value beyond base-only and behavioral impulsivity models. In contrast, none of the behavioral impulsivity measures were significant prospective predictors of substance initiation or perceived harm, nor were they linked to concurrent use or perceived harm.

Therefore, collectively, evidence from this study points to a potential prioritization of impulsivity questionnaires, particularly the CBCL externalizing scale, over behavioral tasks in large-scale, population-based substance use research when resources are limited, or when presenting participants with computerized tasks can be logistically challenging. We do note that because the CBCL externalizing scale and our substance use and perceived-harm outcomes are based on youth or parent report, their shared method variance may have contributed to the stronger predictive performance of questionnaire measures relative to behavioral tasks.

Behavioral impulsivity measures failed to provide additional predictive information beyond demographics in nearly all analyses. This highlights a potential critical limitation of standard metrics derived from behavioral tasks, which have frequently (Yarkoni & Westfall, 2017), as observed here, struggled to capture real-world outcomes. The inability of behavioral impulsivity to predict substance use initiation may reflect shortcomings in the sensitivity of typical summary metrics (aggregate performance across trials) to capture nuances and dynamic processes. Summary measures may obscure meaningful variability from trial to trial or context-specific adaptations, such as changes in impulsivity in response to high-stakes trials (Gilman et al., 2015), peer observation (Weigard et al., 2014), or peer influence (Gilman et al., 2014, 2015). Moreover, behavioral measures of impulsivity, along with computerized cognitive testing more broadly, are often central to large, multi-site developmental studies aimed at issues extending beyond substance use, including brain development and other healthcare outcomes. Similarly, more sensitive longitudinal assessments and/or new measurement approaches for behavioral tasks (e.g. trial-by-trial computational modeling) may be necessary to uncover significant predictive patterns related to substance use. For instance, parameters derived from computational modeling of successive deck choices in the Iowa Gambling Task demonstrated stronger relationships with drug use metrics than the typical crude tally of risky deck choices (Ahn et al., 2014). Finally, it is worth noting that the primary outcomes in this context (substance use and perceived harm of substance use) were themselves evaluated through questionnaires. The increased predictive utility of questionnaires may thus reflect shared measurement error between questionnaires/self-reports, and conversely, the difficulties faced by behavioral tasks in predicting substance use may highlight broader issues in multi-method agreement within psychology (e.g. the poor correspondence between questionnaire and behavioral task impulsivity measures).

Our findings emphasize the importance of leveraging consortia and large population datasets for studying adolescent substance use risk and etiology. Large sample sizes (*n* > 1600) substantially improve the reproducibility of impulsivity measures that predict substance use outcomes. The combination of relatively modest effect sizes and small samples, particularly when substance use is rare (cf., Figure 5), increases the likelihood of false negatives and the overestimation of effect sizes due to sampling bias, which leads to irreproducible findings. Using large consortia data also provides a foundation for careful post hoc confound control (cf., Methods). In the absence of consortia, these results suggest that pooling data across studies may improve the reproducibility of impulsivity-substance use associations. Smaller, single investigator samples are more appropriate when substance use prevalence is high (cf., Figure 5), when samples are carefully matched for relevant confounders, and they remain essential for hypothesis generation, mechanistic exploration, or identifying preliminary trends that can be validated in larger consortia. Smaller samples are also well-positioned to leverage emerging study designs with deep phenotyping and precision longitudinal designs (e.g. EMA, wearables). Advanced statistical techniques (e.g. Bayesian inference) can also help bridge analyses between single-investigator samples and consortia (and vice versa).

The current project boasts several strengths, including the use of a large consortia sample with multi-method and multi-domain measures of impulsivity to predict two adolescent substance use outcomes: initiation and perceived harm. These analyses are conducted in pre-established discovery and validation samples and follow specific guidelines for exploratory analyses. Our findings indicate that while questionnaire-based impulsivity, but not behavioral impulsivity, predicts substance use initiation and perceived harm, the predictive utility remains modest, suggesting considerable room for improvement in predicting substance use outcomes. Furthermore, despite impulsivity’s prominence in neurodevelopmental and etiological models of substance use, these results suggest impulsivity plays a minor role in the complex real-world developmental pathways to substance use. The limited predictive power of impulsivity suggests that other psychological, social, or environmental factors significantly influence substance use outcomes. Although it is central to neurodevelopment and etiological models, focusing solely on impulsivity may obscure broader and more complex influences on adolescent substance use outcomes and other health-compromising behaviors (Green et al., 2024).

Our longitudinal analyses emphasize that the concurrent associations between impulsivity and substance use are more robust than the prospective prediction of future substance use based on earlier impulsivity. This may help explain why large, cross-sectional differences in impulsivity among individuals with and without substance use are often observed (Leeman et al., 2014); however, these relationships tend to be small or inconsistent in longitudinal studies (Ernst et al., 2006). Integrating these observations, impulsivity may be more closely linked to substance use following initiation, such as during regular use or progression to dependence.

This analysis also revealed several limitations. First, the very young age (9–10) of the baseline sample may not be ideal for certain items, such as those in assessments like the UPPS-P, which was generally developed for older adolescents and adults. In contrast, the parent-report CBCL (which showed the most relationships) was developed in the context of mental health symptoms earlier in childhood. Second, the ABCD cohort, while recruited to match the racial composition of American 9–10-year-olds in 2015 (Garavan et al., 2018) is largely a self-selected sample of higher-functioning families, with parent education, income, and other SES factors above the US average. Though we worked to carefully account for these factors as covariates in our analyses, future work should extend these findings to broader more diverse samples. A further limitation is that both predictors and outcomes were assessed through report-based measures. Substance use was measured via adolescent self-report, which is the gold standard in large-scale epidemiological cohorts (e.g. Monitoring the Future) but may introduce shared method variance when paired with questionnaire predictors such as the CBCL. While biological assays (e.g. hair toxicology) could provide complementary validation, these methods are not sufficiently sensitive to detect early, low-level initiation that was the focus of this study. Thus, reliance on self- and parent-report remains the most practical and validated approach at this developmental stage. Finally, although the CBCL emerged as the most robust predictor, it is important to note that the CBCL-E is broader than a ‘pure’ impulsivity measure, as it captures a range of transdiagnostic externalizing and maladaptive behaviors (e.g. oppositionality, rule-breaking) in addition to disinhibition.

In conclusion, our findings show that although questionnaire-based impulsivity measures statistically outperform behavioral tasks, the *absolute* predictive capability for early substance use initiation is low, as even the best-performing model yielded a PPV of only 0.28%, meaning that nearly all adolescents identified as ‘high risk’ would not go on to initiate use. This pattern underscores a central point: large samples can detect statistically reliable associations that have little translational value. As the ABCD cohort ages and variability in exposure increases, it will be important to examine whether impulsivity becomes more predictive of escalation or problematic use. A key area for future exploration is leveraging these results to test theories of impulsivity in later stages of substance use.

## Supporting information

10.1017/S0033291726103225.sm001 Gilman et al. supplementary material Gilman et al. supplementary material

## Supplementary material

The supplementary material for this article can be found at http://doi.org/10.1017/S0033291726103225.

## Presentation information

We submitted a preregistration to the Open Science Framework on 21 June 2022. It is available at https://osf.io/fq4vz. Please see the Supplementary Information for deviations from the preregistration.
