# Supplementary material for: Impulsivity-related predictors of adolescent substance use initiation
Source: Psychol Med. 2026 Feb 6;56:e41. doi: 10.1017/S0033291726103225 (PMC12890431; doi:10.1017/S0033291726103225)
Supplement: Gilman et al. supplementary material [file S0033291726103225sup001.docx]

**Impulsivity-Related Predictors of Adolescent Substance Use Initiation**

**Supplemental Materials**

Table of Contents

[Supplemental methods 3](#_Toc213837358)

[Supplemental results 7](#_Toc213837359)

[Supplemental Figure 1 7](#_Toc213837360)

[Supplemental Figure 2 8](#_Toc213837361)

[Supplemental Figure 3 9](#_Toc213837362)

[Supplemental Figure 4 10](#_Toc213837363)

[Supplemental Figure 5 11](#_Toc213837364)

[Supplemental Table 1.1 12](#_Toc213837365)

[Supplemental Table 1.2 13](#_Toc213837366)

[Supplemental Table 2.1 14](#_Toc213837367)

[Supplemental Table 2.2 15](#_Toc213837368)

[Supplemental Table 2.3 16](#_Toc213837369)

[Supplemental Table 2.4 17](#_Toc213837370)

[Supplemental Table 2.5 18](#_Toc213837371)

[Supplemental Table 3.1 19](#_Toc213837372)

[Supplemental Table 3.2 20](#_Toc213837373)

[Supplemental Table 3.3 21](#_Toc213837374)

[Supplemental Table 3.4 22](#_Toc213837375)

[Supplemental Table 3.5 23](#_Toc213837376)

[Supplemental Table 4.1 24](#_Toc213837377)

[Supplemental Table 4.2 25](#_Toc213837378)

[Supplemental Table 4.3 26](#_Toc213837379)

[Supplemental Table 4.4 27](#_Toc213837380)

[Supplemental Table 4.5 28](#_Toc213837381)

[Supplemental Table 5.1 29](#_Toc213837382)

[Supplemental Table 5.2 30](#_Toc213837383)

[Supplemental Table 5.3 31](#_Toc213837384)

[Supplemental Table 5.4 32](#_Toc213837385)

[Supplemental Table 5.5 33](#_Toc213837386)

[References 34](#_Toc213837387)

# Supplemental methods

*Participants*: The original sample of 11,868 participants were split into two groups, a discovery half and a validation half, using the reproducible matched samples defined by Feczko et al. (2021). There were 341 participants excluded (36 from site 22, 304 from a third group Feczko et al. defined for template building/model testing, and 1 not found in the groups defined by Feczko et al.). Our final sample therefore consisted of 5,770 in our discovery group and 5,757 in our validation group. All data were from ABCD release version 5.1.

*Outcomes*: We followed the approach of Sullivan et al. (2022) when creating our substance use outcome. We defined four broad categories of substance use type (alcohol, nicotine/tobacco, cannabis, other), with three categories of use in order of severity (none, experimentation, initiation). We focus on initiation only for this paper. Alcohol initiation was based on endorsement of the ‘full drink of beer, wine, or liquor (rum, vodka, gin, whiskey)’ item. Nicotine/tobacco initiation was based on any endorsement of the ‘more than just a puff’ items for tobacco cigarettes or electronic cigarettes/vape pens/e-hookahs or ‘ever tried’ items for smokeless tobacco/chew, cigars/traditional cigars/little cigars/cigarillos, pipes, or hookahs. Cannabis initiation was based on endorsement of the ’more than just a puff’ item for marijuana/pot/weed/ganja or ‘ever tried’ items for blunts, edible marijuana/pot cookies/gummy bears/brownies, marijuana oils/concentrates, marijuana infused alcohol drinks, or concentrated marijuana tinctures. Other substance use initiation was based on endorsement of the ‘ever tried’ items for synthetic MJ/K2/spice, commercial liquids/sprays/gases, cough syrup/tranquilizers/opioids/Vicodin, cocaine, bath salts, methamphetamines/meth/crystal meth, amphetamines/ecstasy/molly/MDMA, ketamine, GBH, hallucinogens, psychedelic mushrooms, salvia, or steroids.

We then defined a single binary outcome for any substance use initiation by year 3, using a carry-forward approach. If a participant reported initiation for any of the four categories defined above during any of the yearly time points (baseline to year 3), they were marked has having substance use initiation. The any substance use initiation outcome was set to missing for participants who had missing data for all four substance use categories and no substance use initiation prior to year 3.

We also examined summed scores from the perceived harms inventory examining participants’ perception of the risks people expose themselves to when using substances. Individual items asked participants to rate the risk of harm from consuming certain amounts of a substance using a 0 (no risk) to 3 (great risk) rating. We created separate subscale scores for alcohol (summing over 3 items, with scores ranging from 0 – 9), nicotine/tobacco (summing over 5 items, with scores ranging from 0 – 15), and cannabis (summing over 3 items, with scores ranging from 0 – 9). We then defined a total score, the sum over the 3 subscale scores.

*Predictors*: Our primary predictors of interest were measures of impulsivity. We examined two types of impulsivity measures, self-report (i.e., summed score from standardized questionnaire measures) and behavioral (i.e., summary scores from cognitive tasks). We looked at 3 self-report measures of impulsivity: (1) the Urgency-Premeditation-Perseverance-Sensation Seeking-Positive Urgency inventory (UPPS-P), (2) the Behavioral Activation Scale (BAS), and the Child Behavior Checklist (CBCL). We additionally looked at several demographic measures. Predictors were split into 3 sets:

1. **Set A [Base]** - Variables (included in all models), consisting of 11 measures collected at baseline: Family type (single child [referent], siblings, twins/triplets), age in years, biological sex (male [referent] or female), race (White [referent], Black or African American, collapsed, multiple races), ethnicity (Hispanic/Latino: no [referent] or yes), combined income (between $50,000 to $99,000 [referent], less than $50,000, over $100,000, not provided), highest combined education (Bachelor/Associate degree [referent], less than a high school diploma, high school diploma/GED, some college, post-graduate degree), marriage and employment type (married – both in labor force [referent], married – one in labor force, married – none in labor force, other – both in labor force, other – one in labor force, other – lone parent in labor force, other – none in labor force), parental history of substance use (no [referent] or yes), parental history of mental health issues (depression, mania, paranoia, counseling, nervous breakdown, counseling, hospitalized for mental health; no [referent] or yes), parental history of issues with job or law enforcement [referent] or yes).
2. **Set B [Questionnaire-based impulsivity]** - The questionnaire-based measures of impulsivity, collected at baseline and year 2: the 5 subscales of the UPPS-P, Negative Urgency (NU), Premeditation (PR), Perseverance (PE), Sensation Seeking (SS), and Positive Urgency (PU); the 3 subscales of the BAS, Drive (DR), Fun Seeking (FS), and Reward Responsiveness (RR); and one modified subscale from the CBCL, Externalizing (E) with items pertaining to substance use removed.
3. **Set C [Behavioral-based impulsivity]** - The behavioral measures of impulsivity, collected at baseline and year 2 (or year 1 and year 3 for delayed discounting): the summary score from the delay discounting task (DDT), the estimate of the log of the delay discounting rate, or log(k); the summary score from the stop-signal task (SST), the estimate of the stop signal reaction time (SSRT); the raw score from the NIH toolbox flanker task (FT).

The collapsed racial category combined over selection of American Indian/Alaska Native, Asian, Native Hawaiian/other Pacific Islander, and other. We obtained the log of the delay discounting rate by fitting Mazur’s (1987) one parameter model to the delay discounting cognitive task, as per Kohler, Lichenstein, and Yip (2022). Estimates of the stop signal reaction time were provided by Alexander Weigard from fits of the RDEX-ABCD model to participants’ stop signal task data (see Weigard, Matzke, Tanis, & Heathcote, 2023).

*Imputation*: Patterns of missingness were complex, with missing data both for predictors and outcomes. Missing data was therefore imputed 48 times using predictive mean matching as implemented in the R package ‘mice’ (version 3.16.0; van Buuren & Groothuis-Oudshoorn, 2011). The imputation model used all predictors as well as the any substance use initiation and perceived harm alcohol, nicotine/tobacco, and cannabis subscales.

*Analyses*: First, we examined descriptive summaries of substance use initiation and average ratings for perceived harms, using only non-missing data across years 0 – 3 (the perceived harms questionnaire was only queried post-baseline) We looked at the proportions for substance use initiation over years 0 to 3 for alcohol, nicotine/tobacco, cannabis, and any substance use including the other category. We included 95% uncertainty intervals using Jefferys’ interval for binomial proportions. We looked at the average rating (over a response scale of 0 to 3) for the perceived harms subscales on alcohol, nicotine/tobacco, and cannabis, as well as the total score combining over these subscales. We included 95% uncertainty intervals based on the standard one-sample t-test.

We computed correlations between (a) impulsivity measures and (b) outcomes. As many of the impulsivity measures exhibited distinctly non-normal distribution, and given substance use initiation was a categorical variable, we used the Kendall rank correlation coefficient. Correlation coefficients were computed across the 48 imputations and then pooled according to Rubin’s rules.

We also created predictive models of our outcomes. We used a multilevel logistic regression model to predict substance use initiation by year 3 and used a multilevel binomial regression model (Kahn & Raftery, 1996) to predict summed scores for the perceived harms subscales and total score at year 3. We used binomial regression for the perceived harm scores to account (a) for how scores were integer values bounded between 0 and an upper limit, and (b) for how scores exhibited a high degree of negative skew (most scores close to the upper limit with a long tail towards 0). All models included a site-varying intercept and demographic variables (Set A predictors).

We fit 4 different models: (1) a base model (Set A predictors), (2) a base and questionnaire-based impulsivity model (Set A and B predictors), (3) a base and behavioral-based impulsivity model (Set A and C predictors), and (4) a base and questionnaire and behavioral-based impulsivity model with all predictors (Set A, B, and C), also called the full model. Models were fit separately to each imputation and estimates for a model were then pooled according to Rubin’s rules.

We identified predictors of substance use initiation and perceived harms via two approaches. First, we checked whether predictors in the full model fit to the discovery data were statistically significant at p < .05 after adjusting for multiple comparisons via the Benjamini-Hochberg method. We then confirmed that these predictors remained statistically significant by refitting the model to the validation data, thereby checking replicability of our results. Second, we assessed predictive performance of the models by assessing how well model fits to the discovery data predicted outcomes from the validation data. The ability of the discovery-based model to predict validation data was evaluated using area under the curve (AUC), using DeLong’s test (DeLong, DeLong, & Clarke-Pearson, 1988) to determine if models (a) performed significantly better than an intercept-only model with AUC of 0.5 and (b) performed significantly better compared to each other. For convenience and ease of interpretation, we computed AUC for the binomial regression applied to perceived harms by converting the summed scores to a binary outcome via median split. As a sensitivity analyses, we also examined two metrics of predictive performance for substance use initiation: (1) positive predictive value (PPV; also known as precision), the proportion of predicted instances of substance use initiation that corresponded with actual substance use initiation (i.e., true positives / (true positives + false positives), and (2) recall, the proportion of actual substance use initiation the model correctly predicted as substance use initiation (i.e., true positives / ( true positives + false negatives). Similarly, we also examined an additional metric of predictive performance for the perceived harms outcome: mean-square error (MSE), the average of the square of the difference between the predicted and observed perceived harms score. For PPV, recall, and MSE, statistical significance was determined via a paired samples t-test applied to the underlying proportions of correct categorization and differences between predicted and observed, respectively.

*Power*: Using the estimates from our predictive model, we also ran a power analysis to provide guidance for researchers. We used a resampling approach to generate realistic data from the ABCD data. Specifically, we generated 2,016 resampled training (n=5,770) and testing (n=5,757) data sets by randomly sampling with replacement rows from the respective discovery and validation imputed data sets. For each of the 2,016 data sets we simulated binary outcome data and refit it, estimating power via a Monte Carlo approach. The generating model parameters were set to the estimates from the full model predicting substance use initiation in the discovery data, sans any random effects structure. We refit simulated outcomes across different sample sizes (50 – 5,000) for (a) “univariate” models with only one of the self-report/behavioral impulsivity measures, and (b) a multivariate model with all 6 impulsivity measures. After repeating this process for all 2,016 resampled data sets, we computed power (proportion of iterations with p < .05) for (1) odds ratios for the impulsivity measures from the multivariate model, and (2) AUC compared to an intercept-only model for both the “univariate” and multivariate model.

# Supplemental results

## Supplemental Figure 1

Breakdown of division of participants in original full sample into discovery and validation sets using the splits devised by Feczko et al. (2021), as well as exclusions.

## Supplemental Figure 2

**A**. Correlation heatmap using Kendall’s τ for concurrent (years 2-3) impulsivity measures in the discovery data set. Correlations with an absolute magnitude exceeding a pre-established threshold of .08 are shown in color (positive correlations: red, negative correlations: blue). Correlations below this threshold are shown in white. **B.** Absolute magnitude of discovery correlations (1) among questionnaire (Quest.) impulsivity (Imp.) measures, (2) between behavioral (Beh.) and questionnaire measures, and (3) among behavioral measures. **C**. Correlation heatmap using Kendall’s τ for concurrent (years 2-3) impulsivity measures in the validation data set. **D.** Absolute magnitude of validation correlations (1) among questionnaire (Quest.) impulsivity (Imp.) measures, (2) between behavioral (Beh.) and questionnaire measures, and (3) among behavioral measures.


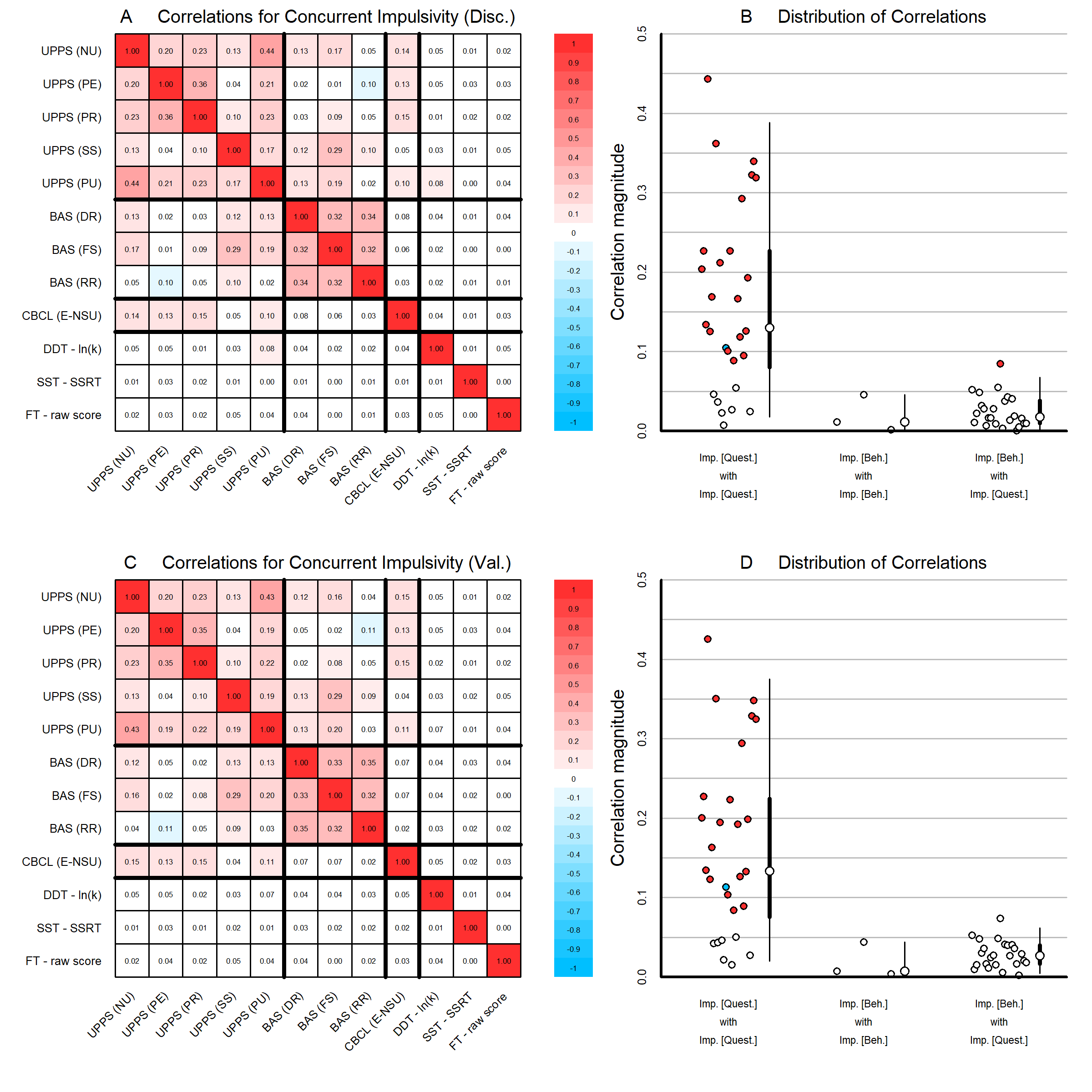


## Supplemental Figure 3

**A**. Odds ratios and 95% uncertainty intervals for each demographic measure from the baseline full model predicting any substance use initiation by year 3 (Circles represent estimates from the model fitted to the discovery data, while triangles denote estimates from the model fitted to the validation data; filled symbols indicate estimates with FDR-adjusted p < .05). **B.** Odds ratios and 95% uncertainty intervals for each demographic measure from the concurrent full model predicting any substance use initiation by year 3. **C**. Change in percent total and 95% uncertainty intervals for each demographic measure from the baseline full model predicting total perceived harms at year 3 **D.** Change in percent total and 95% uncertainty intervals for each demographic measure from the concurrent full model predicting total perceived harms at year 3.


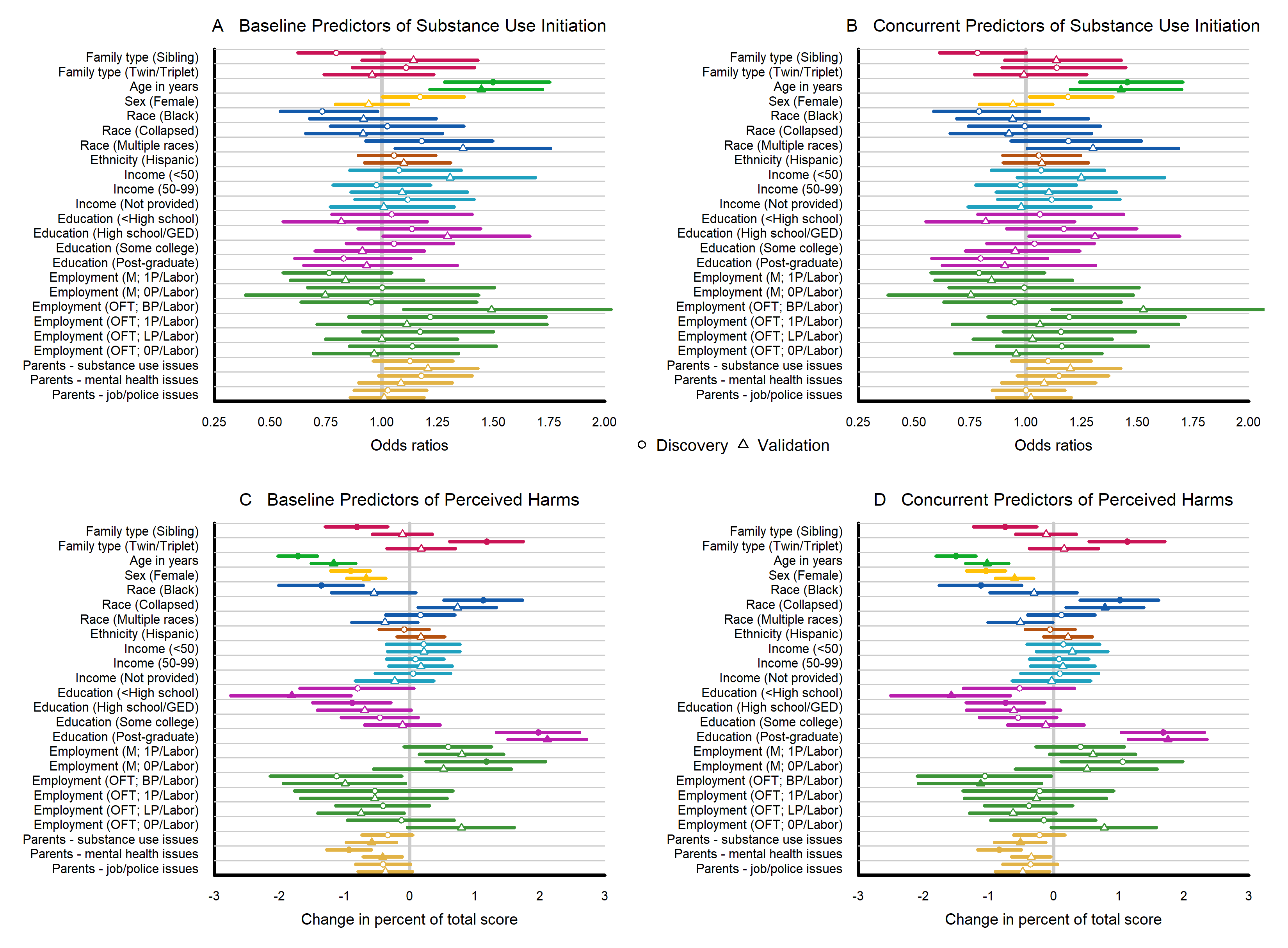


## Supplemental Figure 4

Differences in predictive performance evaluated via 10-fold cross-validation for area under the curve (AUC), positive predictive value (PPV), and recall of the Base model versus the Base model + Impulsivity [Questionnaire] (blue), the Base model + Impulsivity [Behavioral] (Orange), and the Full model (Green) when predicting year 3 substance use. Top panels are for models using predictors measured at year 0/1, bottom panels are for predictors measured at year 2/3. Boxplots are the min, 1^st^ quartile, median, 3rd quartile, and max for the 10 metrics produced via cross-validation, dots and error bars are the associated mean and 95% uncertainty interval obtain via the standard error and t-distribution.


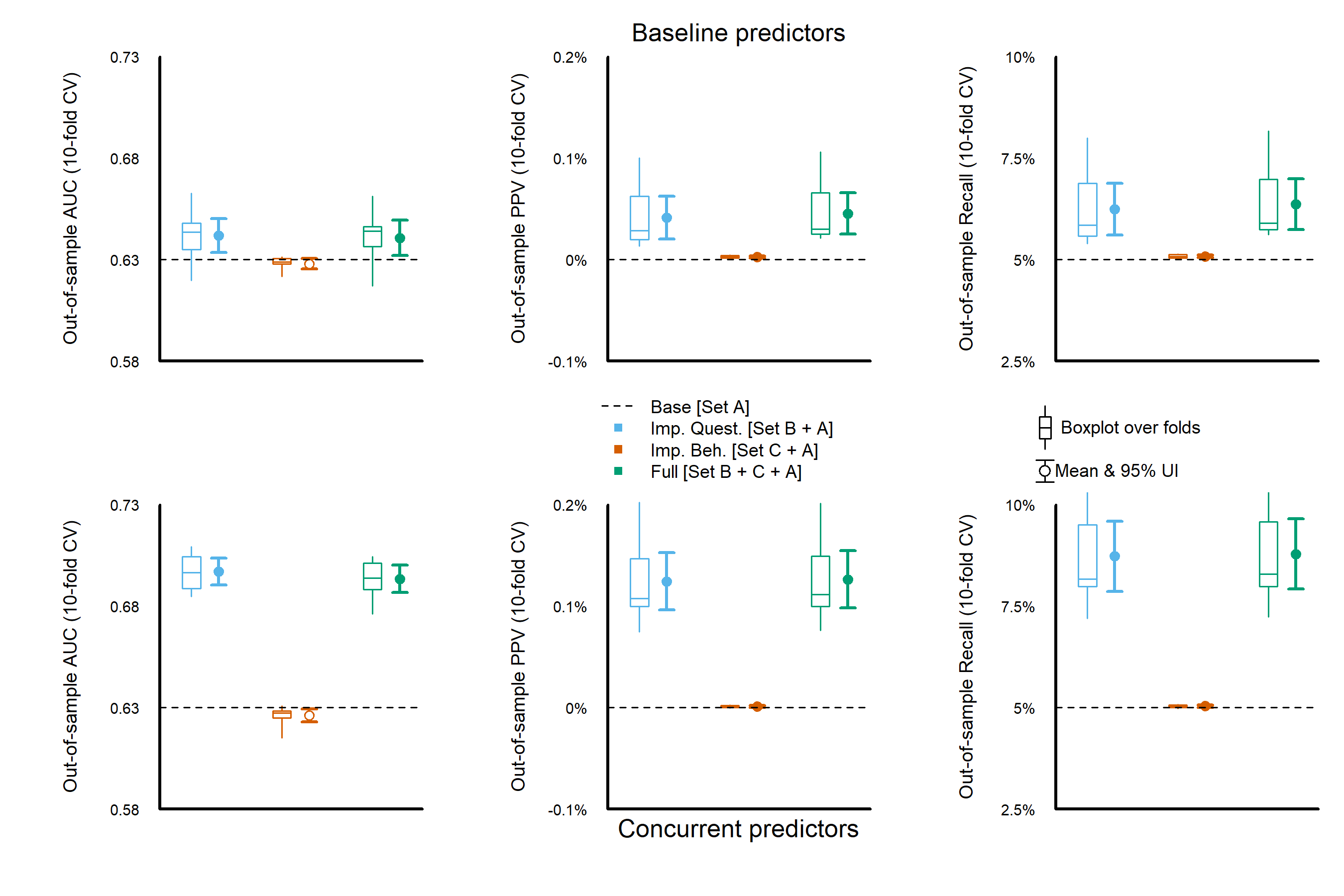


## Supplemental Figure 5

Differences in predictive performance evaluated via 10-fold cross-validation for area under the curve following a median split(AUC) and mean-square error (MSE) of the Base model versus the Base model + Impulsivity [Questionnaire] (blue), the Base model + Impulsivity [Behavioral] (Orange), and the Full model (Green) when predicting year 3 total perceived harms score. Top panels are for models using predictors measured at year 0/1, bottom panels are for predictors measured at year 2/3. Boxplots are the min, 1^st^ quartile, median, 3rd quartile, and max for the 10 metrics produced via cross-validation, dots and error bars are the associated mean and 95% uncertainty interval obtain via the standard error and t-distribution.


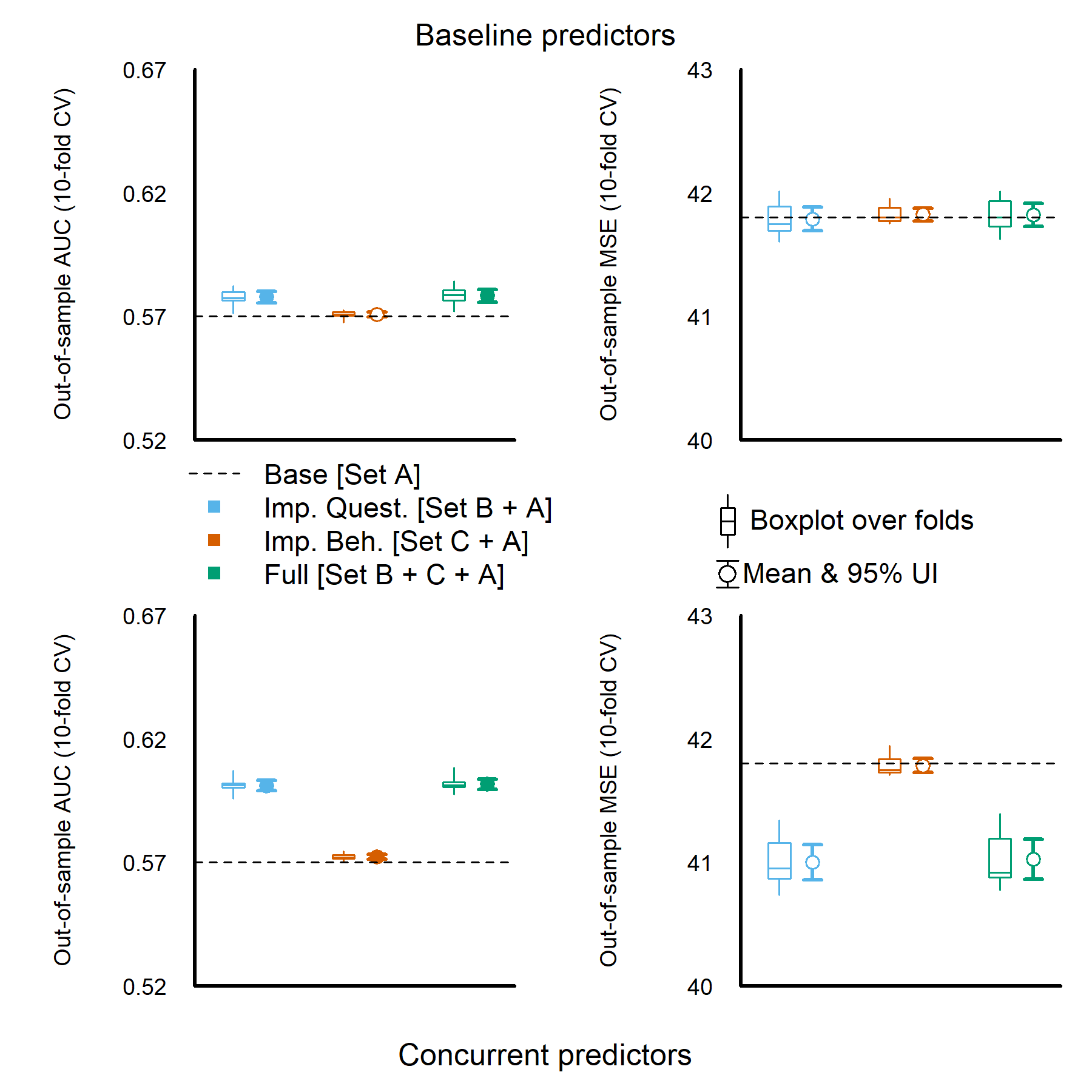


## Supplemental Table 1.1

Descriptives and percentage imputed for demographics by arm.

|  | Discovery arm | | Validation arm | |
| --- | --- | --- | --- | --- |
|  | Statistics | Imputed; % (n) | Statistics | Imputed; % (n) |
| Sample size | 5770 |  | 5757 |  |
| Participant characteristics |  |  |  |  |
| --- Sibling status |  |  |  |  |
| ------ Single child; % yes (n) | 65.9% (3803) |  | 65.4% (3765) |  |
| ------ Sibling; % yes (n) | 15.4% (886) |  | 15.8% (912) |  |
| ------ Twin/triplet; % yes (n) | 18.7% (1081) |  | 18.8% (1080) |  |
| --- Age in years; M (SD) | 9.9 (0.6) |  | 9.9 (0.6) |  |
| --- Biological sex |  |  |  |  |
| ------ Female; % yes (n) | 48.4% (2791) |  | 47.2% (2715) |  |
| ------ Male; % yes (n) | 51.6% (2979) |  | 52.8% (3042) |  |
| --- Race |  |  |  |  |
| ------ Black/African American; % yes (n) | 15.7% (905) |  | 16.3% (936) |  |
| ------ Collapsed; % yes (n) | 7.1% (410) |  | 7.3% (422) |  |
| ------ Multiple races; % yes (n) | 12.0% (690) |  | 12.8% (737) |  |
| ------ White; % yes (n) | 65.2% (3765) |  | 63.6% (3662) |  |
| --- Ethnicity |  |  |  |  |
| ------ Hispanic/Latinx; % yes (n) | 20.6% (1190) | 1.5% (84) | 20.7% (1191) | 1.1% (84) |
| ------ Not Hispanic/Latinx; % yes (n) | 79.4% (4580) |  | 79.3% (4566) |  |
| --- Combined parental income |  |  |  |  |
| ------ < $50k; % yes (n) | 26.7% (1542) | 0.0% (0) | 27.3% (1573) | 0.0% (0) |
| ------ $50k - $99k; % yes (n) | 26.2% (1514) |  | 25.7% (1478) |  |
| ------ > $100k; % yes (n) | 38.5% (2221) |  | 38.5% (2215) |  |
| ------ Not provided; % yes (n) | 8.5% (493) |  | 8.5% (490) |  |
| --- Highest parental education |  |  |  |  |
| ------ Some high school; % yes (n) | 5.2% (300) | 0.2% (10) | 4.8% (278) | 0.1% (10) |
| ------ High school diploma/GED; % yes (n) | 9.0% (520) |  | 9.9% (569) |  |
| ------ Some college; % yes (n) | 12.5% (721) |  | 12.9% (745) |  |
| ------ Bachelor/Associate degree; % yes (n) | 39.2% (2262) |  | 38.2% (2197) |  |
| ------ Post-graduate degree; % yes (n) | 34.1% (1967) |  | 34.2% (1968) |  |
| --- Family type/Employment |  |  |  |  |
| ------ Married/Both in labor force; % yes (n) | 42.4% (2445) | 0.6% (32) | 42.8% (2466) | 1.0% (32) |
| ------ Married/One in labor force; % yes (n) | 23.2% (1338) |  | 21.9% (1260) |  |
| ------ Married/No one in labor force; % yes (n) | 2.4% (140) |  | 2.4% (141) |  |
| ------ Other/Both in labor force; % yes (n) | 3.4% (198) |  | 2.8% (160) |  |
| ------ Other/One in labor force; % yes (n) | 2.1% (123) |  | 2.5% (143) |  |
| ------ Other/Lone parent in labor force; % yes (n) | 19.4% (1120) |  | 19.8% (1139) |  |
| ------ Other/No one in labor force; % yes (n) | 7.0% (406) |  | 7.8% (449) |  |
| --- Parents with prior substance use issues |  |  |  |  |
| ------ No issues; % yes (n) | 80.9% (4670) | 2.5% (144) | 79.1% (4555) | 2.3% (144) |
| ------ Prior issues; % yes (n) | 19.1% (1100) |  | 20.9% (1202) |  |
| --- Parents with prior mental health issues |  |  |  |  |
| ------ No prior issues; % yes (n) | 52.9% (3050) | 1.7% (96) | 51.6% (2969) | 1.7% (96) |
| --- Prior issues; % yes (n) | 47.1% (2720) |  | 48.4% (2788) |  |
| --- Parents with prior job/law enforcement issues |  |  |  |  |
| ------ No prior issues; % yes (n) | 86.9% (5016) | 3.1% (176) | 85.7% (4936) | 3.0% (176) |
| ------ Prior issues; % yes (n) | 13.1% (754) |  | 14.3% (821) |  |

## Supplemental Table 1.2

Descriptives and percentage imputed for impulsivity measures by year and arm.

|  | Discovery arm | | Validation arm | |
| --- | --- | --- | --- | --- |
|  | Statistics | Imputed; % (n) | Statistics | Imputed; % (n) |
| Impulsivity [Self-report] |  |  |  |  |
| --- UPPS-P [Baseline] |  |  |  |  |
| ------ Negative Urgency; M (SD) | 8.4 (2.6) | 0.2% (11) | 8.5 (2.7) | 0.2% (11) |
| ------ Perseverance; M (SD) | 7.0 (2.2) |  | 7.0 (2.3) |  |
| ------ Premeditation; M (SD) | 7.8 (2.4) |  | 7.7 (2.4) |  |
| ------ Sensation Seeking; M (SD) | 9.7 (2.7) |  | 9.8 (2.7) |  |
| ------ Positive Urgency; M (SD) | 8.0 (2.9) |  | 8.0 (3.0) |  |
| --- UPPS-P [Year 2] |  |  |  |  |
| ------ Negative Urgency; M (SD) | 7.8 (2.3) | 7.8% (449) | 7.8 (2.4) | 7.7% (449) |
| ------ Perseverance; M (SD) | 7.0 (2.2) |  | 7.0 (2.3) |  |
| ------ Premeditation; M (SD) | 7.8 (2.2) |  | 7.8 (2.3) |  |
| ------ Sensation Seeking; M (SD) | 9.5 (2.7) |  | 9.5 (2.7) |  |
| ------ Positive Urgency; M (SD) | 7.4 (2.7) |  | 7.4 (2.7) |  |
| --- BAS [Baseline] |  |  |  |  |
| ------ BAS - Drive; M (SD) | 4.1 (3.1) | 0.2% (9) | 4.2 (3.1) | 0.2% (9) |
| ------ BAS - Fun Seeking; M (SD) | 5.7 (2.6) |  | 5.7 (2.7) |  |
| ------ BAS - Reward Responsiveness; M (SD) | 11.0 (2.9) |  | 11.0 (2.9) |  |
| --- BAS [Year 2] |  |  |  |  |
| ------ BAS - Drive; M (SD) | 3.7 (2.8) | 7.8% (449) | 3.7 (2.8) | 7.7% (449) |
| ------ BAS - Fun Seeking; M (SD) | 4.5 (2.6) |  | 4.6 (2.6) |  |
| ------ BAS - Reward Responsiveness; M (SD) | 9.9 (3.1) |  | 9.9 (3.1) |  |
| --- CBCL [Baseline] |  |  |  |  |
| ------ Externalizing; M (SD) | 4.4 (5.9) |  | 4.5 (5.8) |  |
| --- CBCL [Year 2] |  |  |  |  |
| ------ Externalizing; M (SD) | 4.0 (5.7) |  | 3.9 (5.5) |  |
| --- Delay discounting task [Year 1] |  |  |  |  |
| ------ ln(k); M (SD) | -1.20 (2.17) | 52.6% (3037) | -1.15 (2.20) | 53.0% (3037) |
| --- Delay discounting task [Year 3] |  |  |  |  |
| ------ ln(k); M (SD) | -1.47 (2.03) | 57.4% (3314) | -1.44 (2.03) | 59.3% (3314) |
| --- Stop signal task [Baseline] |  |  |  |  |
| ------ SSRT; M (SD) | 0.261 (0.032) | 32.5% (1878) | 0.262 (0.032) | 30.7% (1878) |
| --- Stop signal task [Year 2] |  |  |  |  |
| ------ SSRT; M (SD) | 0.238 (0.031) | 47.7% (2750) | 0.239 (0.030) | 48.4% (2750) |
| --- Flanker task [Baseline] |  |  |  |  |
| ------ Raw score; M (SD) | 7.7 (0.9) | 1.2% (71) | 7.7 (0.9) | 1.3% (71) |
| --- Flanker task [Year 2] |  |  |  |  |
| ------ Raw score; M (SD) | 8.3 (0.8) | 30.6% (1767) | 8.3 (0.8) | 31.2% (1767) |

## Supplemental Table 2.1

Effect sizes (odds ratios) and p-values (FDR adjusted) for baseline impulsivity measures from the comprehensive multivariate logistic regression model predicting year 3 substance use initiation fit to the discovery arm data.

|  |  | P-value | |
| --- | --- | --- | --- |
| Term | Effect size (95% UI) | Unadjusted | Adjusted |
| UPPS (NU) | 0.99 (0.84 to 1.18) | p = 0.944 | p = 0.995 |
| UPPS (PE) | 1.03 (0.88 to 1.22) | p = 0.691 | p = 0.982 |
| UPPS (PR) | 1.24 (1.05 to 1.45) | p = 0.010 | p = 0.088 |
| UPPS (SS) | 1.17 (1.00 to 1.38) | p = 0.047 | p = 0.252 |
| UPPS (PU) | 1.03 (0.87 to 1.23) | p = 0.728 | p = 0.982 |
| BAS (DR) | 1.00 (0.84 to 1.19) | p = 0.983 | p = 0.995 |
| BAS (FS) | 1.01 (0.85 to 1.22) | p = 0.876 | p = 0.982 |
| BAS (RR) | 1.08 (0.90 to 1.30) | p = 0.398 | p = 0.760 |
| DDT - ln(k) | 1.04 (0.84 to 1.28) | p = 0.743 | p = 0.982 |
| SST - SSRT | 0.95 (0.80 to 1.13) | p = 0.581 | p = 0.935 |
| FT - raw score | 1.01 (0.86 to 1.18) | p = 0.911 | p = 0.991 |

## Supplemental Table 2.2

Effect sizes (odds ratios) and p-values (FDR adjusted) for demographic measures from the comprehensive multivariate logistic regression model predicting year 3 substance use initiation fit to the discovery arm data.

|  |  | P-value | |
| --- | --- | --- | --- |
| Term | Effect size (95% UI) | Unadjusted | Adjusted |
| Family type (Sibling) | 0.79 (0.62 to 1.01) | p = 0.063 | p = 0.292 |
| Family type (Twin/Triplet) | 1.11 (0.87 to 1.41) | p = 0.411 | p = 0.760 |
| Age in years | 1.50 (1.28 to 1.75) | p < 0.001 | p < 0.001 |
| Sex (Female) | 1.17 (1.00 to 1.37) | p = 0.048 | p = 0.252 |
| Race (Black) | 0.73 (0.55 to 0.98) | p = 0.037 | p = 0.252 |
| Race (Collapsed) | 1.02 (0.77 to 1.37) | p = 0.867 | p = 0.982 |
| Race (Multiple races) | 1.18 (0.93 to 1.50) | p = 0.177 | p = 0.547 |
| Ethnicity (Hispanic) | 1.05 (0.89 to 1.24) | p = 0.528 | p = 0.889 |
| Income (<50) | 1.08 (0.86 to 1.35) | p = 0.525 | p = 0.889 |
| Income (50-99) | 0.98 (0.78 to 1.22) | p = 0.828 | p = 0.982 |
| Income (Not provided) | 1.11 (0.88 to 1.41) | p = 0.370 | p = 0.760 |
| Education (<High school) | 1.04 (0.78 to 1.40) | p = 0.774 | p = 0.982 |
| Education (High school/GED) | 1.13 (0.89 to 1.44) | p = 0.301 | p = 0.695 |
| Education (Some college) | 1.05 (0.84 to 1.32) | p = 0.650 | p = 0.982 |
| Education (Post-graduate) | 0.83 (0.61 to 1.13) | p = 0.234 | p = 0.618 |
| Employment (M; 1P/Labor) | 0.76 (0.56 to 1.04) | p = 0.090 | p = 0.335 |
| Employment (M; 0P/Labor) | 1.00 (0.67 to 1.50) | p = 0.995 | p = 0.995 |
| Employment (OFT; BP/Labor) | 0.95 (0.64 to 1.42) | p = 0.815 | p = 0.982 |
| Employment (OFT; 1P/Labor) | 1.22 (0.85 to 1.74) | p = 0.282 | p = 0.695 |
| Employment (OFT; LP/Labor) | 1.17 (0.91 to 1.50) | p = 0.212 | p = 0.603 |
| Employment (OFT; 0P/Labor) | 1.14 (0.86 to 1.51) | p = 0.376 | p = 0.760 |
| Parents - substance use issues | 1.13 (0.96 to 1.32) | p = 0.144 | p = 0.483 |
| Parents - mental health issues | 1.18 (0.99 to 1.40) | p = 0.072 | p = 0.295 |
| Parents - job/police issues | 1.03 (0.88 to 1.20) | p = 0.750 | p = 0.982 |

## Supplemental Table 2.3

Effect sizes (odds ratios) and p-values (FDR adjusted) for baseline impulsivity measures from the comprehensive multivariate logistic regression model predicting year 3 substance use initiation fit to the validation arm data.

|  |  | P-value | |
| --- | --- | --- | --- |
| Term | Effect size (95% UI) | Unadjusted | Adjusted |
| UPPS (NU) | 1.15 (0.95 to 1.39) | p = 0.149 | p = 0.425 |
| UPPS (PE) | 1.01 (0.84 to 1.21) | p = 0.906 | p = 0.958 |
| UPPS (PR) | 1.13 (0.95 to 1.34) | p = 0.174 | p = 0.460 |
| UPPS (SS) | 1.22 (1.03 to 1.46) | p = 0.025 | p = 0.130 |
| UPPS (PU) | 1.03 (0.86 to 1.24) | p = 0.732 | p = 0.902 |
| BAS (DR) | 1.03 (0.84 to 1.25) | p = 0.800 | p = 0.925 |
| BAS (FS) | 1.18 (0.97 to 1.43) | p = 0.090 | p = 0.302 |
| BAS (RR) | 0.77 (0.64 to 0.94) | p = 0.009 | p = 0.077 |
| DDT - ln(k) | 1.13 (0.90 to 1.41) | p = 0.302 | p = 0.621 |
| SST - SSRT | 0.85 (0.69 to 1.06) | p = 0.142 | p = 0.425 |
| FT - raw score | 1.02 (0.87 to 1.21) | p = 0.784 | p = 0.925 |

## Supplemental Table 2.4

Effect sizes (odds ratios) and p-values (FDR adjusted) for demographic measures from the comprehensive multivariate logistic regression model predicting year 3 substance use initiation fit to the validation arm data.

|  |  | P-value | |
| --- | --- | --- | --- |
| Term | Effect size (95% UI) | Unadjusted | Adjusted |
| Family type (Sibling) | 1.14 (0.91 to 1.43) | p = 0.249 | p = 0.615 |
| Family type (Twin/Triplet) | 0.96 (0.74 to 1.23) | p = 0.727 | p = 0.902 |
| Age in years | 1.45 (1.21 to 1.72) | p < 0.001 | p < 0.001 |
| Sex (Female) | 0.94 (0.79 to 1.12) | p = 0.491 | p = 0.773 |
| Race (Black) | 0.92 (0.68 to 1.24) | p = 0.577 | p = 0.851 |
| Race (Collapsed) | 0.92 (0.66 to 1.27) | p = 0.598 | p = 0.851 |
| Race (Multiple races) | 1.36 (1.06 to 1.76) | p = 0.016 | p = 0.099 |
| Ethnicity (Hispanic) | 1.10 (0.92 to 1.31) | p = 0.291 | p = 0.621 |
| Income (<50) | 1.31 (1.01 to 1.69) | p = 0.041 | p = 0.167 |
| Income (50-99) | 1.09 (0.86 to 1.38) | p = 0.468 | p = 0.773 |
| Income (Not provided) | 1.01 (0.77 to 1.32) | p = 0.950 | p = 0.976 |
| Education (<High school) | 0.82 (0.56 to 1.20) | p = 0.309 | p = 0.621 |
| Education (High school/GED) | 1.29 (1.01 to 1.66) | p = 0.045 | p = 0.167 |
| Education (Some college) | 0.91 (0.70 to 1.19) | p = 0.501 | p = 0.773 |
| Education (Post-graduate) | 0.93 (0.65 to 1.34) | p = 0.702 | p = 0.902 |
| Employment (M; 1P/Labor) | 0.84 (0.59 to 1.19) | p = 0.319 | p = 0.621 |
| Employment (M; 0P/Labor) | 0.75 (0.39 to 1.43) | p = 0.379 | p = 0.702 |
| Employment (OFT; BP/Labor) | 1.49 (1.10 to 2.03) | p = 0.010 | p = 0.077 |
| Employment (OFT; 1P/Labor) | 1.11 (0.71 to 1.74) | p = 0.643 | p = 0.882 |
| Employment (OFT; LP/Labor) | 1.00 (0.75 to 1.34) | p = 1.000 | p = 1.000 |
| Employment (OFT; 0P/Labor) | 0.97 (0.69 to 1.34) | p = 0.835 | p = 0.936 |
| Parents - substance use issues | 1.21 (1.02 to 1.43) | p = 0.031 | p = 0.144 |
| Parents - mental health issues | 1.09 (0.90 to 1.32) | p = 0.400 | p = 0.704 |
| Parents - job/police issues | 1.01 (0.86 to 1.19) | p = 0.906 | p = 0.958 |

## Supplemental Table 2.5

Predictive performance consisting of area under the curve (AUC), positive predictive value (PPV), and recall for model fit to discovery set predicting validation set with pairwise comparisons (difference, standard error, and p-value following FDR correction) between reduced models (Set A - base predictors; Set A + B - base and questionnaire-based impulsivity predictors; Set A + C - base and behavioral-based impulsivity measures) and the full model (Set A + B + C - base and both questionnaire and behavioral-based impulsivity measures) using baseline measures to predict year 3 substance use.

| Model | Metric | Base + Imp. [Quest.] | Base + Imp. [Beh.] | Base + Imp. [Quest. + Beh.] |
| --- | --- | --- | --- | --- |
|  | AUC | 0.712 | 0.690 | 0.715 |
| Base | 0.688 | 0.025 (0.01); p = 0.024 | 0.002 (0.004); p = 0.534 | 0.028 (0.011); p = 0.024 |
| Base + Imp. [Quest.] | 0.712 |  | -0.022 (0.01); p = 0.048 | 0.003 (0.004); p = 0.534 |
| Base + Imp. [Beh.] | 0.690 |  |  | 0.025 (0.01); p = 0.024 |
|  | PPV | 0.21% | 0.17% | 0.21% |
| Base | 0.17% | 0.04 (0.01); p = 0.001 | 0.00 (0); p = 0.458 | 0.04 (0.01); p = 0.001 |
| Base + Imp. [Quest.] | 0.21% |  | -0.03 (0.01); p = 0.002 | 0.00 (0); p = 0.458 |
| Base + Imp. [Beh.] | 0.17% |  |  | 0.04 (0.01); p = 0.001 |
|  | Recall | 6.72% | 5.60% | 6.79% |
| Base | 5.55% | 1.17 (0.32); p = 0.001 | 0.05 (0.07); p = 0.462 | 1.24 (0.34); p = 0.001 |
| Base + Imp. [Quest.] | 6.72% |  | -1.12 (0.33); p = 0.001 | 0.07 (0.09); p = 0.462 |
| Base + Imp. [Beh.] | 5.60% |  |  | 1.19 (0.33); p = 0.001 |

## Supplemental Table 3.1

Effect sizes (odds ratios) and p-values (FDR adjusted) for concurrent impulsivity measures from the comprehensive multivariate logistic regression model predicting year 3 substance use initiation fit to the discovery arm data.

|  |  | P-value | |
| --- | --- | --- | --- |
| Term | Effect size (95% UI) | Unadjusted | Adjusted |
| UPPS (NU) | 1.18 (0.99 to 1.40) | p = 0.059 | p = 0.217 |
| UPPS (PE) | 1.20 (1.01 to 1.42) | p = 0.038 | p = 0.176 |
| UPPS (PR) | 1.11 (0.94 to 1.31) | p = 0.232 | p = 0.480 |
| UPPS (SS) | 1.40 (1.16 to 1.67) | p < 0.001 | p = 0.003 |
| UPPS (PU) | 1.01 (0.85 to 1.20) | p = 0.881 | p = 0.988 |
| BAS (DR) | 1.00 (0.83 to 1.20) | p = 0.976 | p = 1.000 |
| BAS (FS) | 1.35 (1.12 to 1.63) | p = 0.001 | p = 0.011 |
| BAS (RR) | 0.82 (0.68 to 0.99) | p = 0.037 | p = 0.176 |
| DDT - ln(k) | 1.02 (0.82 to 1.27) | p = 0.870 | p = 0.988 |
| SST - SSRT | 1.04 (0.86 to 1.26) | p = 0.670 | p = 0.919 |
| FT - raw score | 1.12 (0.93 to 1.35) | p = 0.251 | p = 0.480 |

## Supplemental Table 3.2

Effect sizes (odds ratios) and p-values (FDR adjusted) for demographic measures from the comprehensive multivariate logistic regression model predicting year 3 substance use initiation fit to the discovery arm data.

|  |  | P-value | |
| --- | --- | --- | --- |
| Term | Effect size (95% UI) | Unadjusted | Adjusted |
| Family type (Sibling) | 0.78 (0.61 to 1.00) | p = 0.051 | p = 0.210 |
| Family type (Twin/Triplet) | 1.14 (0.89 to 1.45) | p = 0.298 | p = 0.518 |
| Age in years | 1.46 (1.24 to 1.70) | p < 0.001 | p < 0.001 |
| Sex (Female) | 1.19 (1.01 to 1.39) | p = 0.032 | p = 0.176 |
| Race (Black) | 0.79 (0.59 to 1.06) | p = 0.118 | p = 0.392 |
| Race (Collapsed) | 1.00 (0.74 to 1.33) | p = 0.973 | p = 1.000 |
| Race (Multiple races) | 1.19 (0.93 to 1.52) | p = 0.162 | p = 0.405 |
| Ethnicity (Hispanic) | 1.06 (0.90 to 1.24) | p = 0.509 | p = 0.753 |
| Income (<50) | 1.07 (0.85 to 1.35) | p = 0.574 | p = 0.817 |
| Income (50-99) | 0.98 (0.78 to 1.22) | p = 0.828 | p = 0.988 |
| Income (Not provided) | 1.11 (0.87 to 1.42) | p = 0.380 | p = 0.586 |
| Education (<High school) | 1.06 (0.78 to 1.44) | p = 0.698 | p = 0.922 |
| Education (High school/GED) | 1.17 (0.91 to 1.50) | p = 0.212 | p = 0.480 |
| Education (Some college) | 1.04 (0.82 to 1.31) | p = 0.751 | p = 0.958 |
| Education (Post-graduate) | 0.80 (0.58 to 1.10) | p = 0.164 | p = 0.405 |
| Employment (M; 1P/Labor) | 0.79 (0.58 to 1.08) | p = 0.145 | p = 0.405 |
| Employment (M; 0P/Labor) | 0.99 (0.65 to 1.51) | p = 0.975 | p = 1.000 |
| Employment (OFT; BP/Labor) | 0.95 (0.63 to 1.43) | p = 0.803 | p = 0.988 |
| Employment (OFT; 1P/Labor) | 1.19 (0.83 to 1.72) | p = 0.339 | p = 0.546 |
| Employment (OFT; LP/Labor) | 1.16 (0.90 to 1.49) | p = 0.260 | p = 0.480 |
| Employment (OFT; 0P/Labor) | 1.16 (0.87 to 1.55) | p = 0.308 | p = 0.518 |
| Parents - substance use issues | 1.10 (0.93 to 1.29) | p = 0.252 | p = 0.480 |
| Parents - mental health issues | 1.15 (0.96 to 1.37) | p = 0.127 | p = 0.392 |
| Parents - job/police issues | 1.00 (0.85 to 1.18) | p = 1.000 | p = 1.000 |

## Supplemental Table 3.3

Effect sizes (odds ratios) and p-values (FDR adjusted) for concurrent impulsivity measures from the comprehensive multivariate logistic regression model predicting year 3 substance use initiation fit to the validation arm data.

|  |  | P-value | |
| --- | --- | --- | --- |
| Term | Effect size (95% UI) | Unadjusted | Adjusted |
| UPPS (NU) | 1.35 (1.11 to 1.64) | p = 0.003 | p = 0.029 |
| UPPS (PE) | 1.13 (0.93 to 1.37) | p = 0.206 | p = 0.587 |
| UPPS (PR) | 1.08 (0.88 to 1.31) | p = 0.465 | p = 0.787 |
| UPPS (SS) | 1.29 (1.06 to 1.57) | p = 0.010 | p = 0.060 |
| UPPS (PU) | 1.07 (0.88 to 1.29) | p = 0.511 | p = 0.787 |
| BAS (DR) | 1.20 (0.99 to 1.46) | p = 0.068 | p = 0.253 |
| BAS (FS) | 1.08 (0.87 to 1.33) | p = 0.479 | p = 0.787 |
| BAS (RR) | 0.84 (0.69 to 1.04) | p = 0.106 | p = 0.328 |
| DDT - ln(k) | 1.07 (0.85 to 1.35) | p = 0.563 | p = 0.834 |
| SST - SSRT | 1.02 (0.83 to 1.25) | p = 0.840 | p = 0.899 |
| FT - raw score | 1.04 (0.87 to 1.24) | p = 0.684 | p = 0.882 |

## Supplemental Table 3.4

Effect sizes (odds ratios) and p-values (FDR adjusted) for demographic measures from the comprehensive multivariate logistic regression model predicting year 3 substance use initiation fit to the validation arm data.

|  |  | P-value | |
| --- | --- | --- | --- |
| Term | Effect size (95% UI) | Unadjusted | Adjusted |
| Family type (Sibling) | 1.14 (0.91 to 1.43) | p = 0.269 | p = 0.712 |
| Family type (Twin/Triplet) | 0.99 (0.77 to 1.27) | p = 0.941 | p = 0.941 |
| Age in years | 1.43 (1.20 to 1.70) | p < 0.001 | p = 0.001 |
| Sex (Female) | 0.94 (0.79 to 1.12) | p = 0.498 | p = 0.787 |
| Race (Black) | 0.94 (0.69 to 1.28) | p = 0.691 | p = 0.882 |
| Race (Collapsed) | 0.93 (0.66 to 1.30) | p = 0.654 | p = 0.882 |
| Race (Multiple races) | 1.30 (1.00 to 1.68) | p = 0.046 | p = 0.189 |
| Ethnicity (Hispanic) | 1.07 (0.90 to 1.28) | p = 0.438 | p = 0.787 |
| Income (<50) | 1.25 (0.96 to 1.62) | p = 0.094 | p = 0.316 |
| Income (50-99) | 1.10 (0.87 to 1.41) | p = 0.427 | p = 0.787 |
| Income (Not provided) | 0.98 (0.74 to 1.29) | p = 0.877 | p = 0.902 |
| Education (<High school) | 0.82 (0.55 to 1.22) | p = 0.325 | p = 0.787 |
| Education (High school/GED) | 1.31 (1.01 to 1.69) | p = 0.038 | p = 0.184 |
| Education (Some college) | 0.95 (0.73 to 1.24) | p = 0.715 | p = 0.882 |
| Education (Post-graduate) | 0.90 (0.62 to 1.31) | p = 0.596 | p = 0.849 |
| Employment (M; 1P/Labor) | 0.85 (0.59 to 1.21) | p = 0.360 | p = 0.787 |
| Employment (M; 0P/Labor) | 0.75 (0.38 to 1.48) | p = 0.409 | p = 0.787 |
| Employment (OFT; BP/Labor) | 1.53 (1.12 to 2.09) | p = 0.008 | p = 0.058 |
| Employment (OFT; 1P/Labor) | 1.06 (0.67 to 1.68) | p = 0.798 | p = 0.895 |
| Employment (OFT; LP/Labor) | 1.03 (0.76 to 1.39) | p = 0.850 | p = 0.899 |
| Employment (OFT; 0P/Labor) | 0.95 (0.68 to 1.34) | p = 0.789 | p = 0.895 |
| Parents - substance use issues | 1.20 (1.01 to 1.42) | p = 0.040 | p = 0.184 |
| Parents - mental health issues | 1.08 (0.89 to 1.31) | p = 0.422 | p = 0.787 |
| Parents - job/police issues | 1.02 (0.87 to 1.20) | p = 0.786 | p = 0.895 |

## Supplemental Table 3.5

Predictive performance consisting of area under the curve (AUC), positive predictive value (PPV), and recall for model fit to discovery set predicting validation set with pairwise comparisons (difference, standard error, and p-value following FDR correction) between reduced models (Set A - base predictors; Set A + B - base and questionnaire-based impulsivity predictors; Set A + C - base and behavioral-based impulsivity measures) and the full model (Set A + B + C - base and both questionnaire and behavioral-based impulsivity measures) using concurrent measures to predict year 3 substance use.

| Model | Metric | Base + Imp. [Quest.] | Base + Imp. [Beh.] | Base + Imp. [Quest. + Beh.] |
| --- | --- | --- | --- | --- |
|  | AUC | 0.760 | 0.687 | 0.760 |
| Base | 0.688 | 0.073 (0.017); p < 0.001 | -0.001 (0.005); p = 0.978 | 0.073 (0.017); p < 0.001 |
| Base + Imp. [Quest.] | 0.760 |  | -0.073 (0.018); p < 0.001 | 0.000 (0.004); p = 0.978 |
| Base + Imp. [Beh.] | 0.687 |  |  | 0.073 (0.017); p < 0.001 |
|  | PPV | 0.28% | 0.17% | 0.28% |
| Base | 0.17% | 0.11 (0.02); p < 0.001 | 0.00 (0); p = 0.757 | 0.11 (0.02); p < 0.001 |
| Base + Imp. [Quest.] | 0.28% |  | -0.11 (0.02); p < 0.001 | 0.00 (0); p = 0.757 |
| Base + Imp. [Beh.] | 0.17% |  |  | 0.11 (0.02); p < 0.001 |
|  | Recall | 9.02% | 5.58% | 9.10% |
| Base | 5.55% | 3.47 (0.69); p < 0.001 | 0.03 (0.11); p = 0.757 | 3.54 (0.69); p < 0.001 |
| Base + Imp. [Quest.] | 9.02% |  | -3.44 (0.72); p < 0.001 | 0.07 (0.16); p = 0.757 |
| Base + Imp. [Beh.] | 5.58% |  |  | 3.51 (0.7); p < 0.001 |

## Supplemental Table 4.1

Effect sizes (change in percent total) and p-values (FDR adjusted) for baseline impulsivity measures from the comprehensive multivariate binomial regression model predicting year 3 total perceived harms fit to the discovery arm data.

|  |  | P-value | |
| --- | --- | --- | --- |
| Term | Effect size (95% UI) | Unadjusted | Adjusted |
| UPPS (NU) | -0.005 (-0.008 to -0.002) | p = 0.002 | p = 0.006 |
| UPPS (PE) | -0.008 (-0.012 to -0.004) | p < 0.001 | p < 0.001 |
| UPPS (PR) | -0.005 (-0.008 to -0.001) | p = 0.005 | p = 0.014 |
| UPPS (SS) | -0.009 (-0.012 to -0.006) | p < 0.001 | p < 0.001 |
| UPPS (PU) | -0.003 (-0.007 to 0.001) | p = 0.124 | p = 0.177 |
| BAS (DR) | 0.003 (-0.001 to 0.006) | p = 0.135 | p = 0.186 |
| BAS (FS) | -0.005 (-0.009 to -0.001) | p = 0.009 | p = 0.021 |
| BAS (RR) | 0.004 ( 0.000 to 0.007) | p = 0.048 | p = 0.089 |
| DDT - ln(k) | -0.007 (-0.013 to 0.000) | p = 0.040 | p = 0.078 |
| SST - SSRT | -0.005 (-0.010 to 0.000) | p = 0.036 | p = 0.074 |
| FT - raw score | 0.002 (-0.002 to 0.005) | p = 0.314 | p = 0.401 |

## Supplemental Table 4.2

Effect sizes (change in percent total) and p-values (FDR adjusted) for demographic measures from the comprehensive multivariate binomial regression model predicting year 3 total perceived harms fit to the discovery arm data.

|  |  | P-value | |
| --- | --- | --- | --- |
| Term | Effect size (95% UI) | Unadjusted | Adjusted |
| Family type (Sibling) | -0.008 (-0.013 to -0.003) | p = 0.001 | p = 0.003 |
| Family type (Twin/Triplet) | 0.012 ( 0.006 to 0.017) | p < 0.001 | p < 0.001 |
| Age in years | -0.017 (-0.020 to -0.014) | p < 0.001 | p < 0.001 |
| Sex (Female) | -0.009 (-0.012 to -0.006) | p < 0.001 | p < 0.001 |
| Race (Black) | -0.014 (-0.020 to -0.007) | p < 0.001 | p < 0.001 |
| Race (Collapsed) | 0.011 ( 0.005 to 0.017) | p < 0.001 | p = 0.001 |
| Race (Multiple races) | 0.002 (-0.004 to 0.007) | p = 0.542 | p = 0.627 |
| Ethnicity (Hispanic) | -0.001 (-0.005 to 0.003) | p = 0.657 | p = 0.736 |
| Income (<50) | 0.002 (-0.004 to 0.008) | p = 0.462 | p = 0.552 |
| Income (50-99) | 0.001 (-0.004 to 0.005) | p = 0.697 | p = 0.759 |
| Income (Not provided) | 0.001 (-0.005 to 0.006) | p = 0.863 | p = 0.887 |
| Education (<High school) | -0.008 (-0.017 to 0.001) | p = 0.069 | p = 0.117 |
| Education (High school/GED) | -0.009 (-0.015 to -0.003) | p = 0.004 | p = 0.011 |
| Education (Some college) | -0.005 (-0.011 to 0.001) | p = 0.124 | p = 0.177 |
| Education (Post-graduate) | 0.020 ( 0.013 to 0.026) | p < 0.001 | p < 0.001 |
| Employment (M; 1P/Labor) | 0.006 (-0.001 to 0.013) | p = 0.086 | p = 0.132 |
| Employment (M; 0P/Labor) | 0.012 ( 0.002 to 0.021) | p = 0.014 | p = 0.032 |
| Employment (OFT; BP/Labor) | -0.011 (-0.021 to -0.001) | p = 0.028 | p = 0.060 |
| Employment (OFT; 1P/Labor) | -0.005 (-0.018 to 0.007) | p = 0.379 | p = 0.468 |
| Employment (OFT; LP/Labor) | -0.004 (-0.011 to 0.003) | p = 0.257 | p = 0.340 |
| Employment (OFT; 0P/Labor) | -0.001 (-0.010 to 0.007) | p = 0.753 | p = 0.796 |
| Parents - substance use issues | -0.003 (-0.007 to 0.000) | p = 0.084 | p = 0.132 |
| Parents - mental health issues | -0.009 (-0.013 to -0.006) | p < 0.001 | p < 0.001 |
| Parents - job/police issues | -0.004 (-0.008 to 0.000) | p = 0.055 | p = 0.097 |

## Supplemental Table 4.3

Effect sizes (change in percent total) and p-values (FDR adjusted) for baseline impulsivity measures from the comprehensive multivariate binomial regression model predicting year 3 total perceived harms fit to the validation arm data.

|  |  | P-value | |
| --- | --- | --- | --- |
| Term | Effect size (95% UI) | Unadjusted | Adjusted |
| UPPS (NU) | -0.002 (-0.006 to 0.001) | p = 0.169 | p = 0.273 |
| UPPS (PE) | -0.001 (-0.004 to 0.003) | p = 0.614 | p = 0.688 |
| UPPS (PR) | -0.012 (-0.016 to -0.009) | p < 0.001 | p < 0.001 |
| UPPS (SS) | -0.008 (-0.011 to -0.005) | p < 0.001 | p < 0.001 |
| UPPS (PU) | -0.006 (-0.010 to -0.003) | p = 0.001 | p = 0.005 |
| BAS (DR) | 0.000 (-0.003 to 0.004) | p = 0.841 | p = 0.864 |
| BAS (FS) | 0.003 (-0.001 to 0.006) | p = 0.129 | p = 0.231 |
| BAS (RR) | 0.000 (-0.003 to 0.004) | p = 0.920 | p = 0.920 |
| DDT - ln(k) | -0.008 (-0.014 to -0.002) | p = 0.013 | p = 0.044 |
| SST - SSRT | -0.005 (-0.011 to 0.001) | p = 0.131 | p = 0.231 |
| FT - raw score | 0.001 (-0.002 to 0.004) | p = 0.542 | p = 0.626 |

## Supplemental Table 4.4

Effect sizes (change in percent total) and p-values (FDR adjusted) for demographic measures from the comprehensive multivariate binomial regression model predicting year 3 total perceived harms fit to the validation arm data.

|  |  | P-value | |
| --- | --- | --- | --- |
| Term | Effect size (95% UI) | Unadjusted | Adjusted |
| Family type (Sibling) | -0.001 (-0.006 to 0.003) | p = 0.633 | p = 0.688 |
| Family type (Twin/Triplet) | 0.002 (-0.004 to 0.007) | p = 0.505 | p = 0.603 |
| Age in years | -0.012 (-0.015 to -0.008) | p < 0.001 | p < 0.001 |
| Sex (Female) | -0.007 (-0.010 to -0.004) | p < 0.001 | p < 0.001 |
| Race (Black) | -0.006 (-0.012 to 0.001) | p = 0.093 | p = 0.181 |
| Race (Collapsed) | 0.007 ( 0.001 to 0.013) | p = 0.018 | p = 0.052 |
| Race (Multiple races) | -0.004 (-0.009 to 0.001) | p = 0.138 | p = 0.232 |
| Ethnicity (Hispanic) | 0.002 (-0.002 to 0.005) | p = 0.356 | p = 0.488 |
| Income (<50) | 0.002 (-0.003 to 0.008) | p = 0.433 | p = 0.572 |
| Income (50-99) | 0.002 (-0.003 to 0.007) | p = 0.489 | p = 0.603 |
| Income (Not provided) | -0.002 (-0.008 to 0.004) | p = 0.449 | p = 0.572 |
| Education (<High school) | -0.018 (-0.027 to -0.009) | p < 0.001 | p = 0.001 |
| Education (High school/GED) | -0.007 (-0.014 to 0.000) | p = 0.058 | p = 0.131 |
| Education (Some college) | -0.001 (-0.007 to 0.005) | p = 0.709 | p = 0.749 |
| Education (Post-graduate) | 0.021 ( 0.015 to 0.027) | p < 0.001 | p < 0.001 |
| Employment (M; 1P/Labor) | 0.008 ( 0.001 to 0.014) | p = 0.017 | p = 0.052 |
| Employment (M; 0P/Labor) | 0.005 (-0.006 to 0.016) | p = 0.336 | p = 0.488 |
| Employment (OFT; BP/Labor) | -0.010 (-0.019 to -0.001) | p = 0.035 | p = 0.086 |
| Employment (OFT; 1P/Labor) | -0.005 (-0.017 to 0.006) | p = 0.344 | p = 0.488 |
| Employment (OFT; LP/Labor) | -0.007 (-0.014 to -0.001) | p = 0.027 | p = 0.070 |
| Employment (OFT; 0P/Labor) | 0.008 ( 0.000 to 0.016) | p = 0.060 | p = 0.131 |
| Parents - substance use issues | -0.006 (-0.010 to -0.002) | p = 0.003 | p = 0.011 |
| Parents - mental health issues | -0.004 (-0.007 to -0.001) | p = 0.007 | p = 0.025 |
| Parents - job/police issues | -0.004 (-0.008 to 0.000) | p = 0.075 | p = 0.155 |

## Supplemental Table 4.5

Predictive performance consisting of area under the curve (AUC; predicting a median split of scores) and mean-square error (MSE; using raw values) for model fit to discovery set predicting validation set with pairwise comparisons (difference, standard error, and p-value following FDR correction) between reduced models (Set A - base predictors; Set A + B - base and questionnaire-based impulsivity predictors; Set A + C - base and behavioral-based impulsivity measures) and the full model (Set A + B + C - base and both questionnaire and behavioral-based impulsivity measures) using baseline measures to predict total perceived harms at year 3.

| Model | Metric | Base + Imp. [Quest.] | Base + Imp. [Beh.] | Base + Imp. [Quest. + Beh.] |
| --- | --- | --- | --- | --- |
|  | AUC | 0.596 | 0.587 | 0.598 |
| Base | 0.585 | 0.011 (0.004); p = 0.006 | 0.002 (0.003); p = 0.421 | 0.013 (0.004); p = 0.006 |
| Base + Imp. [Quest.] | 0.596 |  | -0.009 (0.004); p = 0.074 | 0.002 (0.002); p = 0.421 |
| Base + Imp. [Beh.] | 0.587 |  |  | 0.011 (0.004); p = 0.006 |
|  | MSE | 40.6 | 40.8 | 40.6 |
| Base | 40.9 | -0.3 (0.1); p = 0.154 | -0.1 (0.1); p = 0.478 | -0.3 (0.2); p = 0.154 |
| Base + Imp. [Quest.] | 40.6 |  | 0.2 (0.2); p = 0.42 | -0.1 (0.1); p = 0.61 |
| Base + Imp. [Beh.] | 40.8 |  |  | -0.2 (0.1); p = 0.192 |

## Supplemental Table 5.1

Effect sizes (change in percent total) and p-values (FDR adjusted) for concurrent impulsivity measures from the comprehensive multivariate binomial regression model predicting year 3 total perceived harms fit to the discovery arm data.

|  |  | P-value | |
| --- | --- | --- | --- |
| Term | Effect size (95% UI) | Unadjusted | Adjusted |
| UPPS (NU) | -0.011 (-0.015 to -0.007) | p < 0.001 | p < 0.001 |
| UPPS (PE) | -0.008 (-0.012 to -0.004) | p < 0.001 | p = 0.001 |
| UPPS (PR) | -0.011 (-0.014 to -0.007) | p < 0.001 | p < 0.001 |
| UPPS (SS) | -0.005 (-0.009 to -0.002) | p = 0.001 | p = 0.004 |
| UPPS (PU) | -0.010 (-0.015 to -0.006) | p < 0.001 | p < 0.001 |
| BAS (DR) | -0.003 (-0.007 to 0.002) | p = 0.217 | p = 0.319 |
| BAS (FS) | -0.004 (-0.009 to 0.000) | p = 0.045 | p = 0.083 |
| BAS (RR) | 0.017 ( 0.013 to 0.020) | p < 0.001 | p < 0.001 |
| DDT - ln(k) | -0.010 (-0.017 to -0.003) | p = 0.007 | p = 0.016 |
| SST - SSRT | -0.004 (-0.009 to 0.002) | p = 0.227 | p = 0.319 |
| FT - raw score | 0.002 (-0.002 to 0.006) | p = 0.326 | p = 0.402 |

## Supplemental Table 5.2

Effect sizes (change in percent total) and p-values (FDR adjusted) for demographic measures from the comprehensive multivariate binomial regression model predicting year 3 total perceived harms fit to the discovery arm data.

|  |  | P-value | |
| --- | --- | --- | --- |
| Term | Effect size (95% UI) | Unadjusted | Adjusted |
| Family type (Sibling) | -0.007 (-0.012 to -0.003) | p = 0.003 | p = 0.006 |
| Family type (Twin/Triplet) | 0.011 ( 0.005 to 0.017) | p < 0.001 | p = 0.001 |
| Age in years | -0.015 (-0.018 to -0.012) | p < 0.001 | p < 0.001 |
| Sex (Female) | -0.010 (-0.013 to -0.007) | p < 0.001 | p < 0.001 |
| Race (Black) | -0.011 (-0.018 to -0.005) | p = 0.001 | p = 0.002 |
| Race (Collapsed) | 0.010 ( 0.004 to 0.016) | p = 0.001 | p = 0.004 |
| Race (Multiple races) | 0.001 (-0.004 to 0.006) | p = 0.648 | p = 0.749 |
| Ethnicity (Hispanic) | -0.001 (-0.004 to 0.003) | p = 0.778 | p = 0.778 |
| Income (<50) | 0.001 (-0.004 to 0.007) | p = 0.597 | p = 0.713 |
| Income (50-99) | 0.001 (-0.004 to 0.005) | p = 0.722 | p = 0.763 |
| Income (Not provided) | 0.001 (-0.005 to 0.007) | p = 0.753 | p = 0.774 |
| Education (<High school) | -0.005 (-0.014 to 0.003) | p = 0.220 | p = 0.319 |
| Education (High school/GED) | -0.007 (-0.013 to -0.001) | p = 0.016 | p = 0.035 |
| Education (Some college) | -0.005 (-0.011 to 0.000) | p = 0.069 | p = 0.121 |
| Education (Post-graduate) | 0.017 ( 0.010 to 0.023) | p < 0.001 | p < 0.001 |
| Employment (M; 1P/Labor) | 0.004 (-0.003 to 0.011) | p = 0.233 | p = 0.319 |
| Employment (M; 0P/Labor) | 0.011 ( 0.001 to 0.020) | p = 0.029 | p = 0.059 |
| Employment (OFT; BP/Labor) | -0.011 (-0.021 to 0.000) | p = 0.041 | p = 0.081 |
| Employment (OFT; 1P/Labor) | -0.002 (-0.014 to 0.009) | p = 0.707 | p = 0.763 |
| Employment (OFT; LP/Labor) | -0.004 (-0.011 to 0.003) | p = 0.264 | p = 0.339 |
| Employment (OFT; 0P/Labor) | -0.002 (-0.010 to 0.006) | p = 0.704 | p = 0.763 |
| Parents - substance use issues | -0.002 (-0.006 to 0.002) | p = 0.265 | p = 0.339 |
| Parents - mental health issues | -0.008 (-0.012 to -0.005) | p < 0.001 | p < 0.001 |
| Parents - job/police issues | -0.004 (-0.008 to 0.001) | p = 0.092 | p = 0.155 |

## Supplemental Table 5.3

Effect sizes (change in percent total) and p-values (FDR adjusted) for concurrent impulsivity measures from the comprehensive multivariate binomial regression model predicting year 3 total perceived harms fit to the validation arm data.

|  |  | P-value | |
| --- | --- | --- | --- |
| Term | Effect size (95% UI) | Unadjusted | Adjusted |
| UPPS (NU) | -0.017 (-0.021 to -0.013) | p < 0.001 | p < 0.001 |
| UPPS (PE) | -0.014 (-0.018 to -0.010) | p < 0.001 | p < 0.001 |
| UPPS (PR) | -0.001 (-0.005 to 0.003) | p = 0.584 | p = 0.676 |
| UPPS (SS) | -0.004 (-0.007 to 0.000) | p = 0.038 | p = 0.077 |
| UPPS (PU) | -0.007 (-0.011 to -0.004) | p < 0.001 | p = 0.001 |
| BAS (DR) | 0.002 (-0.002 to 0.006) | p = 0.344 | p = 0.469 |
| BAS (FS) | -0.008 (-0.012 to -0.004) | p < 0.001 | p = 0.001 |
| BAS (RR) | 0.012 ( 0.008 to 0.016) | p < 0.001 | p < 0.001 |
| DDT - ln(k) | -0.007 (-0.013 to -0.002) | p = 0.012 | p = 0.034 |
| SST - SSRT | -0.001 (-0.007 to 0.004) | p = 0.692 | p = 0.711 |
| FT - raw score | 0.005 ( 0.001 to 0.010) | p = 0.024 | p = 0.053 |

## Supplemental Table 5.4

Effect sizes (change in percent total) and p-values (FDR adjusted) for demographic measures from the comprehensive multivariate binomial regression model predicting year 3 total perceived harms fit to the validation arm data.

|  |  | P-value | |
| --- | --- | --- | --- |
| Term | Effect size (95% UI) | Unadjusted | Adjusted |
| Family type (Sibling) | -0.001 (-0.006 to 0.003) | p = 0.624 | p = 0.679 |
| Family type (Twin/Triplet) | 0.002 (-0.004 to 0.007) | p = 0.560 | p = 0.676 |
| Age in years | -0.010 (-0.014 to -0.007) | p < 0.001 | p < 0.001 |
| Sex (Female) | -0.006 (-0.009 to -0.003) | p < 0.001 | p < 0.001 |
| Race (Black) | -0.003 (-0.010 to 0.004) | p = 0.367 | p = 0.469 |
| Race (Collapsed) | 0.008 ( 0.002 to 0.014) | p = 0.011 | p = 0.033 |
| Race (Multiple races) | -0.005 (-0.010 to 0.000) | p = 0.043 | p = 0.083 |
| Ethnicity (Hispanic) | 0.002 (-0.002 to 0.006) | p = 0.248 | p = 0.367 |
| Income (<50) | 0.003 (-0.003 to 0.008) | p = 0.309 | p = 0.440 |
| Income (50-99) | 0.001 (-0.004 to 0.006) | p = 0.574 | p = 0.676 |
| Income (Not provided) | 0.000 (-0.006 to 0.006) | p = 0.918 | p = 0.918 |
| Education (<High school) | -0.016 (-0.025 to -0.007) | p = 0.001 | p = 0.003 |
| Education (High school/GED) | -0.006 (-0.013 to 0.001) | p = 0.093 | p = 0.149 |
| Education (Some college) | -0.001 (-0.007 to 0.005) | p = 0.683 | p = 0.711 |
| Education (Post-graduate) | 0.018 ( 0.012 to 0.024) | p < 0.001 | p < 0.001 |
| Employment (M; 1P/Labor) | 0.006 (-0.001 to 0.013) | p = 0.074 | p = 0.125 |
| Employment (M; 0P/Labor) | 0.005 (-0.006 to 0.016) | p = 0.355 | p = 0.469 |
| Employment (OFT; BP/Labor) | -0.011 (-0.021 to -0.002) | p = 0.019 | p = 0.049 |
| Employment (OFT; 1P/Labor) | -0.003 (-0.014 to 0.008) | p = 0.624 | p = 0.679 |
| Employment (OFT; LP/Labor) | -0.006 (-0.013 to 0.000) | p = 0.063 | p = 0.112 |
| Employment (OFT; 0P/Labor) | 0.008 ( 0.000 to 0.016) | p = 0.061 | p = 0.112 |
| Parents - substance use issues | -0.005 (-0.009 to -0.001) | p = 0.011 | p = 0.033 |
| Parents - mental health issues | -0.003 (-0.007 to 0.000) | p = 0.025 | p = 0.053 |
| Parents - job/police issues | -0.005 (-0.009 to -0.001) | p = 0.025 | p = 0.053 |

## Supplemental Table 5.5

Predictive performance consisting of area under the curve (AUC; predicting a median split of scores) and mean-square error (MSE; using raw values) for model fit to discovery set predicting validation set with pairwise comparisons (difference, standard error, and p-value following FDR correction) between reduced models (Set A - base predictors; Set A + B - base and questionnaire-based impulsivity predictors; Set A + C - base and behavioral-based impulsivity measures) and the full model (Set A + B + C - base and both questionnaire and behavioral-based impulsivity measures) using baseline measures to predict total perceived harms at year 3.

| Model | Metric | Base + Imp. [Quest.] | Base + Imp. [Beh.] | Base + Imp. [Quest. + Beh.] |
| --- | --- | --- | --- | --- |
|  | AUC | 0.618 | 0.588 | 0.619 |
| Base | 0.585 | 0.033 (0.006); p < 0.001 | 0.003 (0.003); p = 0.461 | 0.034 (0.006); p < 0.001 |
| Base + Imp. [Quest.] | 0.618 |  | -0.030 (0.006); p < 0.001 | 0.001 (0.002); p = 0.691 |
| Base + Imp. [Beh.] | 0.588 |  |  | 0.031 (0.006); p < 0.001 |
|  | MSE | 40.0 | 40.8 | 39.9 |
| Base | 40.9 | -1.0 (0.2); p < 0.001 | -0.1 (0.1); p = 0.449 | -1.0 (0.3); p < 0.001 |
| Base + Imp. [Quest.] | 40.0 |  | 0.9 (0.3); p = 0.002 | 0.0 (0.1); p = 0.747 |
| Base + Imp. [Beh.] | 40.8 |  |  | -0.9 (0.2); p < 0.001 |

# References

DeLong, E. R., DeLong, D. M., & Clarke-Pearson, D. L. (1988). Comparing the areas under two or more correlated receiver operating characteristic curves: A nonparametric approach. *Biometrics*, *44* (3), 837 - 845.

Feczko, E., Conan, G., Marek, S., Tervo-Clemmens, B., Cordova, M., Doyle, O., Earl, E., Perrone, A.8, Sturgeon, D., Klein, R., Harman, G., Kilamovich, D., Hermosillo, R., Miranda-Dominguez, O.14, Adebimpe, A., Bertolero, M., Cieslak, M., Covitz, S., Hendrickson, T., …, Fair, D. A. (2021). Adolescent Brain Cognitive Development (ABCD) community MRI collection and utilities. BioRxiv. https://doi.org/10.1101/2021.07.09.451638.

Kahn, M. J., & Raftery, A. E. (1996). *Journal of the American Statistical Association*, *91* (433), 29 - 41. https://doi.org/10.1080/01621459.1996.10476661.

Kohler, R. J., Lichenstein, S. D., & Yip, S. W. (2022). Hyperbolic discounting rates and risk for problematic alcohol use in youth enrolled in the Adolescent Brain and Cognitive Development study. *Addiction Biology*, *27* (2), 1 - 13. https://doi.org/10.1111/adb.13160.

Mazur, J. E. (1987). An adjusting procedure for studying delayed reinforcement. In M. L. Commons, J. E. Mazur, J. A. Nevin, & H. Rachlin (Eds.), *The effect of delay and intervening events on reinforcement value* (pp. 55-73). Lawrence Erlbaum Associates, Inc.

Sullivan, R. M., Wade, Natasha E., Wallace, A. L., Tapert, S. F., Pelham, W. E., Brown, S. A., Cloak, C. C., Ewing, S. W. F., Madden, P. A. F., Martz, M. E., Ross, J. M., Kaiver, C. M., Wirtz, H. G., Heitzeg, M. M., & Lisdahl, K. M. (2022). Substance use patterns in 9 to 13-year olds: Longitudinal findings from the Adolescent Brain Cognitive Development (ABCD) study. *Drug and Alcohol Dependence Reports*, *5*, 1 - 12. https://doi.org/10.1016/j.dadr.2022.100120.

van Buuren, S., & Groothuis-Oudshoorn, K. (2011). mice: Multivariate imputation by chained equations in R. *Journal of Statistical Software*, *45* (3), 1 - 67. https://doi.org/10.18637/jss.v045.i03.

Weigard, A., Maztke, D., Tanis, C., & Heathcote, A. (2023). A cognitive process modeling framework for the ABCD study stop-signal task. *Developmental Cognitive Neuroscience*, *59*, 1-17. https://doi.org/10.1016/j.dcn.2022.101191.
